# Study of Shellfish Growing Area During Normal Harvesting Periods and Following Wastewater Overflows in an Urban Estuary With Complex Hydrography

**DOI:** 10.1007/s12560-023-09579-8

**Published:** 2024-02-08

**Authors:** Carlos J. A. Campos, Pradip Gyawali, Joanne Hewitt

**Affiliations:** 1 Cawthron Institute, 98 Halifax Street East, Nelson, 7042 New Zealand; 2 Jacobs, 47 Hereford Street Level 2, Wynn Williams Building, Christchurch, 8013 New Zealand; 3 Institute of Environmental Science and Research Limited (ESR), Kenepuru Science Centre, 34 Kenepuru Drive, Kenepuru, Porirua, 5240 New Zealand

**Keywords:** Bivalves, Sewage, Enteric viruses, *E. coli*, Tracers, Environmental risk assessment

## Abstract

Viral testing combined with hydrographic studies is considered standard good practice in determining microbiological impacts on shellfish growing areas following wastewater overflows. In this study, norovirus genogroup I and II, indicators of viral contamination (F-RNA bacteriophage genogroup II (F-RNA GII), crAssphage, pepper mild mottle virus) and *Escherichia coli* were monitored during periods of normal harvesting and following overflows in two commercial shellfish growing areas in Otago Harbour (Aotearoa New Zealand). Dye tracing, drogue tracking and analysis of particle tracking modelling were also undertaken to assess the dispersion, dilution and time of travel of wastewater discharged from a pump station discharge that impacts the growing areas. Norovirus was not detected in any of the 218 shellfish samples tested. PMMoV and crAssphage were more prevalent than F-RNA GII as determined by RT-qPCR. The dye study indicated long residence time of the waters (≥5 days) in the embayment impacted by the discharge. No relationships were found between the concentrations of viral indicators or *E. coli* and wastewater dilution, distance between the discharge and the growing areas or time since the last overflow. For the three spills studied (≤327 m^3^), there was little evidence of microbiological impact on the growing areas. This was likely associated with a deep shipping channel that enhances water flushing in the harbour and reduces contaminant transport to the growing areas. We recommend flexibility in the approach for closure/reopening growing areas impacted by spills, particularly for small duration/volume spills and when norovirus is not present in the community.

**Supplementary Information:**

The online version contains supplementary material available at 10.1007/s12560-023-09579-8.

## Introduction

Farming shellfish in coastal environments remote from wastewater discharges is essential to maintain safety and quality standards and continuity of supply of shellfish products (Brown et al., 2020; Fox et al., 2019). However, many shellfish growing areas are intermittently or permanently closed because of the continued use of the marine environment to discharge wastewater, preventing access to many productive areas (Younger et al., 2022). These impacts have been exacerbated by population growth, urban intensification and climate change (Campos & Lees, 2014; Fleming et al., 2006; Freeman et al., 2019). Pollution impacts from intermittent discharges (e.g. combined sewer overflows, pump station overflows, septic tank discharges) are challenging to manage because the frequency, volume and microbial composition of these discharges vary considerably between events (Flannery et al., 2013; Younger et al., 2022). Evans et al. (2016) reported substantial economic losses (in the order of millions of dollars annually) in shellfish growing areas impacted by wastewater overflows during periods of peak harvest in Machias Bay (Maine, USA). In addition to economic losses attributed to harvest closures, loss of consumer confidence following outbreaks of illness can result in long-lasting effects on the industry (Askew, 2009; Younger et al., 2022). Sustainable management of microbiological risks from wastewater overflows requires fit-for-purpose growing area management options and responses.

Several viral pathogens have been implicated in shellfish-related outbreaks overseas, including hepatitis A and E, norovirus, sapovirus, astrovirus and aichi virus (Bellou et al., 2013; Desdouits et al., 2023; Richards, 2016). However, epidemiological studies identify norovirus as the pathogen of greatest concern globally (Bellou et al., 2013; Qiu et al., 2021; White et al., 2022). Norovirus outbreaks associated with contamination of shellfish growing areas with domestic wastewater have been reported in the literature (Bellou et al., 2013; Fitzgerald et al., 2014; Lodo et al., 2014; Miller et al., 2018). Following the implementation of shellfish sanitation programmes in many countries, illnesses due to bacterial pathogens (e.g. *Salmonella* spp., *Shigella* spp., *Campylobacter* spp.) and viruses have been much less common (Pommepuy et al., 2009).

In Aotearoa New Zealand (NZ), the Ministry for Primary Industries (MPI) require a comprehensive sanitation programme to ensure that shellfish are safe to eat. The Regulated Control Scheme—Bivalve Molluscan Shellfish (RCS-BMS) (MPI, 2022) requires that each shellfish growing area be subject to a sanitary survey and classified according to the results of indicator bacteria monitoring (using faecal coliforms/*Escherichia coli*) of water/shellfish (MPI, 2022). The RCS-BMS also prescribes control measures for dealing with pollution in growing areas, including criteria for identifying and sizing prohibited zones. The regulation contains a requirement that if an Animal Product Officer (APO) has reason to believe that an area has been impacted by a wastewater pollution event, the APO must keep the area closed for 28 days from the date of the end of the event, unless a shellfish specialist determines that a greater or lesser time is required (MPI, 2022). The APO may reopen the growing area when there is evidence that the pollution condition that caused the emergency closure no longer exists and there was sufficient time for the microbiological contaminant that may be present in the shellfish/water to return to background levels (MPI, 2022). The 28-day closure period prescribed in the RCS-BMS is supported by data from laboratory studies in which norovirus and hepatitis A were tested in oysters held in tanks with filtered seawater (30–31 ppt) and later transplanted to a commercial growing area with water temperatures of 14–18 °C. Norovirus-positive samples collected at least 4 weeks after transplantation from the growing area supported the recommendation of a 28-day closure in the regulation (Greening et al., 2002). The criteria for reopening growing areas impacted by spills differ between countries. In the USA, the default closure period is 21 days, but this can be shortened to a minimum of 7 days if samples taken from the impacted area contain F-RNA bacteriophage at <50 pfu/100 g (USFDA & ISSC, 2019). Similar requirements apply in Canada (CFIA, 2022) and Australia (ASQAAC, 2016) where impacted growing areas are closed for at least 7 days after the discharge event ceases (with verification sampling) or at least 21 days (without verification sampling).

*Escherichia coli* is a notoriously a poor indicator of viral contamination (Romalde et al., 2002; Younger et al., 2018) and therefore should not be used in isolation as a base for decisions on the duration of closure periods. In the USA, the National Shellfish Sanitation Program prescribes a male specific coliphage (i.e. F-RNA phage) standard for reopening shellfish growing areas impacted by wastewater overflows (USFDA & ISSC, 2019). In addition to viral and/or viral indicator monitoring, studies show that information on wastewater discharge volumes and data from tracer studies and/or hydrodynamic modelling provide information on the fate and transport of wastewater contamination and can assist management of viral risk in growing areas impacted by continuous discharges (Campos et al., 2017a, 2017b; Winterbourn et al., 2016).

The goal of this study was to determine if wastewater dilution studies combined with viral indicator monitoring can be used to predict norovirus risk and manage the reopening of shellfish growing areas impacted by wastewater overflows. The specific objectives of the study were (1) to determine the prevalence and distribution of human norovirus, and indicators of human viral contamination (F-RNA bacteriophage genogroup II (F-RNA GII), crAssphage, pepper mild mottle virus [PMMoV]) and *E. coli* in shellfish during periods of normal harvesting (baseline) and following wastewater overflows (emergency growing area closures); and (2) to study the relationships between concentrations of microbiological contaminants in shellfish and wastewater dilution or other parameters that could be used to predict microbiological impacts from spills. This study adds to the evidence base on viral contamination in NZ shellfish growing areas and international approaches for closing/reopening growing areas impacted by spills.

## Methods

### Study area

**Fig. 1:**
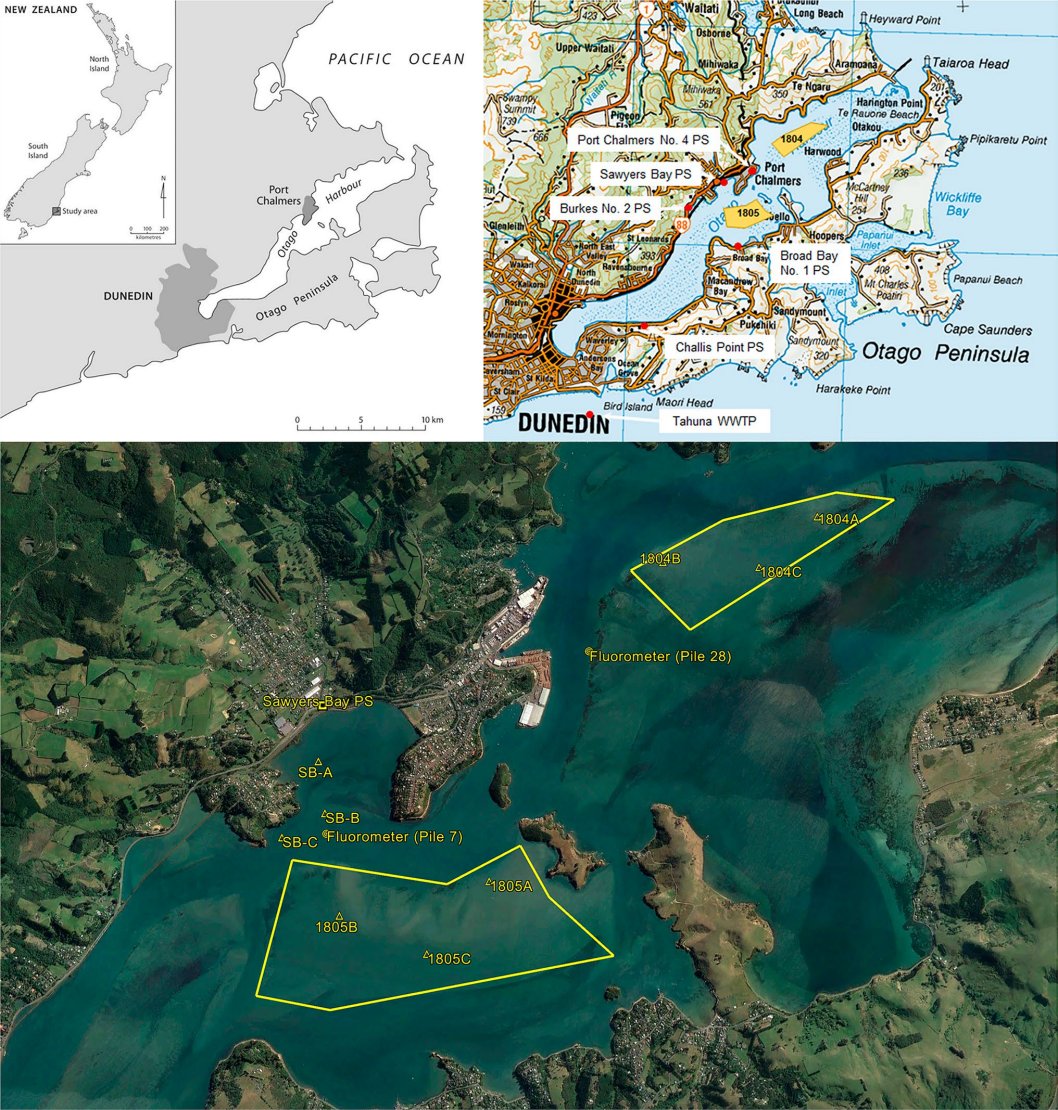
Map of the study area showing the location of the pump station outfalls, the boundaries of the shellfish growing areas, sites where shellfish were deployed in cages (SBA–C), sites where shellfish were hand dredged (1804A–C and 1805A–C) and sites where fluorometers were deployed for the dye study

The study was undertaken in the Otago Harbour on the southeast coast of the South Island of NZ (Fig. 1). The Otago Harbour is a long (21 km), narrow (width at entrance = 1.5 km) and shallow (mean water depth = 4.5 m) tidal inlet with a single channel which meanders in a south-westerly direction from the entrance (Heath, 1976). An artificially maintained shipping channel occurs along the full length of the harbour. The harbour is connected to the ocean via a narrow, deep mouth. At the southwest end of the harbour is the city of Dunedin with a population of ca. 126,000 (Stats NZ, 2023). There are other small settlements in the catchment, including Port Chalmers (population approximately 1,400), Macandrew Bay (population approximately 1,500) and Portobello (population approximately 1,150) (Stats NZ, 2023).

Semi-diurnal tides drive most of the water movements in Otago Harbour, although some variability is also associated with the prevailing southwest and northeast winds (Bell et al., 2009). The mean tidal range at the entrance is 1.5 m (Albrecht & Vennell, 2007). Tidal flows are generally bi-directional, but velocities are stronger on flood than on ebb tides, and the duration of the ebb is longer than that of the flood (Wilson & Sutherland, 1991). At Port Chalmers, peak spring tide velocities reach 0.8 m/s on flood tides and 0.6 m/s on ebb tides (Barnett, 1988). At the harbour entrance, spring ebb and flood currents can peak at 1.3 m/s and 1.5 m/s (Vennell & Old, 1999). The waters in the harbour are well mixed and density gradients are small (Wilson & Sutherland, 1991).

Little neck clams or tuaki (*Austrovenus stutchburyi*) are harvested from the sandbanks at two sites near Port Chalmers: 1804 (0.7 km × 2 km), just to the east of Careys Bay, and 1805 (1 km × 2 km) just to the southeast of Roseneath (Fig. 1). Both shellfish growing areas are centrally located in the harbour. The clams are harvested using ‘body dredges with riddling baskets’ and transported to the company’s factory in Dunedin to supply NZ and overseas markets. In domestic markets, little neck clams are usually sold live and consumed as cooked product. There is no record of illness cases attributed to these shellfish from the Otago Harbour growing areas.

Water quality in the harbour is generally good but can be episodically impacted by stormwater discharges and, occasionally, wastewater overflows. There are no continuous discharges of domestic wastewater to the harbour. Domestic and industrial waste flows from Dunedin and the smaller settlements in the lower catchment are collected and transported for treatment at Tahuna Wastewater Treatment Plant, which discharges tertiary-treated wastewater effluent to the sea via a long sea outfall off the St Kilda coast (Fig. 1). Within the harbour, the sources of greatest viral contamination risk are pump station overflows due to the potentially significant contribution of untreated human waste. The shellfish growing areas have been particularly impacted by overflows from the Sawyers Bay Pump Station (PS), which discharges to Sawyers Bay (approximately 1.2 km from growing area 1805) (Fig. 1). Two other pump stations in the city of Dunedin (Harbour Terrace PS, Marne St PS) discharge to tidal waters or streams and can also affect the microbiological quality of the harbour waters.

### Monitoring approach and wastewater overflows

Concentrations of microbiological contaminants (bacterial faecal indicators, viral human faecal indicators and norovirus) were monitored in samples of little neck clams taken during baseline and emergency growing area closures. Baseline sampling followed the schedule of the official monitoring programme (adverse pollution condition-based sampling regime or, by definition, at times when environmental conditions historically resulted in elevated *E. coli* concentrations; one sample taken each month) during the period December 2020–August 2022. The emergency closure sampling was undertaken at varying time intervals during 28-day closure periods (spill dates are provided in Table 1), depending on the duration of the spills and tides, as follows:

- Spill event 1: days 3, 9 and 18
- Spill event 2: days 2 and 8
- Spill event 3: days 2, 9, 15 and 21.

**Table 1:** Details of wastewater overflows monitored in Otago Harbour. Data provided by Dunedin City Council

Pump station	Approximate distance (m) from the edge of shellfish growing area 1804	Approximate distance (m) from the edge of shellfish growing area 1805	Overflow start date (time)	Overflow end date (time)	Total volume discharged (m^3^)	Cause of overflow	Tide at overflow start time^e^
Spill event 1							
Sawyers Bay PS	4,070	1,200	02/01/2021 (22:32 h)^a^	03/01/2021 (04:07 h)	326.6		Ebb
Burkes No. 2 PS	5,360	1,560	03/01/2021 (19:48 h)	03/01/2021 (22:18 h)	>100 (estimated)	Electrical failure	High water
Spill event 2							
Port Chalmers No 4 (Back Beach) PS	2,000	1,410	01/02/2022^b^	01/02/2022^b^	20 (estimated)	Burst pipe	
Spill event 3							
Challis Point PS	9,660	5,410	13/07/2022 (09:50 h)^c^	14/07/2022 (09:12 h)	997.2	Infiltration of stormwater into pump well due to heavy rainfall	Low water
Broad Bay No. 1 PS	4,760	930	13/07/2022 (21:47 h)	14/07/2022 (00:06 h)	166.8	Infiltration of stormwater into pump well due to heavy rainfall	Low water
Sawyers Bay PS	4,070	1,200	16/07/2022 (05:23 h)^d^	16/07/2022 (08:09 h)	172.7		High water

^a^ Alarm activated on nine occasions. Only total volume provided by DCC. ^b^ No information available on start and end times of the spill. ^c^ Alarm activated on four occasions. ^d^ Alarm activated on six occasions. ^e^ Tidal stage is approximate

Sampling during emergency growing area closures was undertaken following wastewater spill events of varying duration and volume that exceeded the growing area management criterion (150 m^3^). The details of the overflows are summarised in Table 1.

### Sampling sites and sample collection

Little neck clams were sourced from growing area 1805, placed in plastic open-mesh baskets (approximately 11 kg of clams in each basket) and deployed at three sites (identified as SB-A, SB-B and SB-C) in Sawyers Bay (Fig. 1). At these sites, the baskets were suspended from a surface float at approximately 1 m depth above anchor points. These shellfish were deployed in November 2020 and allowed to acclimatise over 2 weeks prior to sampling. In addition to the Sawyers Bay sites, shellfish samples were collected from six sites (three in each growing area; identified as 1804A–C and 1805A–C) during baseline monitoring and emergency closures following wastewater spills from Sawyers Bay PS. At the Sawyers Bay sites, the samples were collected by manually lifting the baskets from a boat. In the classified growing areas, the samples were taken by hand dredging.

Upon collection, the samples were immediately placed in a cool box with ice packs to maintain internal temperature at approximately 4 °C, transported to the Southern Clams factory and sent by courier to Cawthron Analytical for *E. coli* testing by Most Probable Number (MPN) culture method. Upon arrival at Cawthron, sub-samples were taken (whole animals in shell) and immediately sent by overnight courier to ESR for subsequent testing of norovirus genogroups I and II (GI and GII), F-RNA GII, crAssphage and PMMoV by qPCR/reverse-transcription (RT)-qPCR. No more than 24 h elapsed between sample collection and the beginning of *E. coli* testing. At ESR, viral testing was carried out in batches of frozen samples within 2 weeks of sample collection.

### Microbiological testing

*E. coli* testing followed the protocol used in the official monitoring programme (MPI, 2020). Briefly described, this method uses a two-stage, five-tube three-dilution most probable number (MPN) technique to estimate viable numbers of bacteria. The first stage of the method was a resuscitation step consisting of inoculation of Minerals Modified Glutamate Broth (MMGB) with a series of diluted sample homogenates and incubation at 37 ± 1 °C for 24 ± 2 h. The presence of *E. coli* was confirmed by subculturing acid producing tubes onto agar containing 5-bromo-4-chloro-3-indolyl-ß-D glucuronide and detecting ß-glucuronidase activity after incubation at 44 ± 1 °C for 21 ± 3 h. *E. coli* concentrations were determined from the number of confirmed MMGB tubes positive for the bacterium for each dilution tested and the number of positive tubes required for reading the MPN tables. Whole animal homogenates were prepared from the flesh and intravalvular liquid of a minimum of 35 clams per sub-sample and assayed using the MPN method. The results were reported as MPN/100 g of shellfish.

### Virus testing

Shellfish samples were shucked, digestive tissue removed and finely chopped in a Petri dish. The tissue was weighed, and phosphate buffered saline (PBS, pH 7.2) added in a ratio of 2:1 (e.g. 2 mL of PBS added per gram). Murine norovirus (10 µL of 10^5^ genome copies (GC)/mL per gram digestive tissue) was added as a process control. The sample was vortexed for 2 min and 1 mm zirconia/silica beads (dnature diagnostics & research Ltd, Gisborne, NZ) added. The sample was bead-beaten twice at a speed of 6 m/s for 30 s at 20 °C using a FastPrep-24™ homogeniser (MP Biomedicals, CA, US) to further homogenise the tissue. The sample was then centrifuged at 13,000 × g for 2 min and supernatant recovered. This method was selected to avoid compromising any downstream assay that would be used to better inform on infectivity. The viral RNA was then extracted from 200 μL of shellfish homogenate using the Roche High Pure Viral Nucleic Acid Extraction Kit (Roche Molecular Biochemicals Ltd., Mannheim, Germany), according to the manufacturer’s instructions*.*

The presence of norovirus GI and GII, F-RNA GII, PMMoV and murine norovirus was determined by RT-qPCR, and crAssphage by qPCR. Conditions used were as described by Gyawali et al. (2021). qPCR standards were prepared for crAssphage and PMMoV using synthetic gene fragments (gBlocks) (Integrated DNA Technologies, Coralville, LA, US). The pGEM-T Easy Vector System (Promega, Madison, WI, US) was used to generate qPCR plasmid standards for norovirus GI and GII, and F-RNA phage GII. To generate standard curves, gBlock /plasmid standard serial dilutions ranging from 10^6^ to 10^0^ GC/μL were prepared. For CrAssphage qPCR, 20 μL assays consisted of 2.5 μL template DNA, 10 μL of 2 × PerfeCTa qPCR ToughMix (Quantabio, Beverly, MA), final primer and probe concentrations of 600 nM and 300 nM, respectively, and water. For norovirus GI and GII, and F-RNA phage GII RT-qPCR, 25 μL reactions consisted of 2.5 μL RNA, 10 μL qScript XLT 1-step RT-qPCR ToughMix (Quantabio), final primer and probe concentrations of 500 nm and 200 nM for F-RNA phage GII and 400 nM and 200 nM for norovirus GI and GII, and water. Murine norovirus one-step RT-qPCR assays were performed in 20 μL reaction mixtures containing 10 µL qScript XLT 1-step RT-qPCR ToughMix (Quantabio), 2.5 µL nucleic acid, and final primers and probe concentrations of 400 nM and 250 nM, respectively. All qPCR assays were performed in the Bio-Rad CFX96 Touch™ Real-Time PCR Detection System (Bio-Rad Laboratories, Hercules, CA, US). Nucleic acid extractions, mastermix preparation and RT-qPCR/qPCR assays were performed in separate laboratories to minimise cross contamination. An extract blank was included with each batch of nucleic acid extraction. For each RT-qPCR/qPCR batch, positive (DNA standard at 2.5 × 10^4^ GC/reaction) and negative controls (DNase/RNase free water) were included. The Bio-Rad CFX Manager™ Software (Bio-Rad Laboratories) was used to determine the RT-qPCR/qPCR Cq values.

The limit of quantification (LoQ) was defined as the lowest standard concentration detected in all three replicates with a Cq difference less than 2. Using the quantities used for the shellfish processing and the subsequent viral nucleic acid extraction and PCR assay, the LoQ of all targets was 200 GC/gram digestive tissue. Levels below the LoQ were considered as detected but not quantifiable. Where quantifiable, the viral concentrations were expressed as genome copies/gram of digestive tissue.

Murine norovirus recoveries from shellfish digestive tissue were greater than 10%, with no indication of PCR inhibition. Gyawali et al. (2021) reported method sensitivity, specificity and accuracy of F-RNA GII, crAssphage, PMMoV markers for predicting norovirus in shellfish, and therefore, the method was considered appropriate for use in the present study. The F-RNA GII results using RT-qPCR may not be directly comparable with total F-RNA bacteriophage results obtained using a plaque assay, which is the preferred method in the US for managing earlier reopening of growing areas impacted by wastewater spills (USFDA & ISSC, 2019).

### Hydrographic studies

A hydrographic dye tracing and drogue tracking study was undertaken in February 2021 to determine the time of travel and dispersion and the dilution of wastewater contamination from Sawyers Bay PS. Rhodamine WT dye mixed with tap water (approximately 11 L; concentration = 6,948 ppb) was released into Sawyers Bay at a shoreline site 27 m from the Sawyers Bay PS outfall on 10 February 2021. This site was chosen to take advantage of local brackish water flows from a small creek through culvert pipes into Sawyers Bay. These water flows provided good initial mixing for the injected dye mixture thus simulating the conditions observed during an actual wastewater spill occurring on the ebb tide. The dye mixture was injected at constant rate (77 mL/min) using a metering pump FMI Model X for approximately 3 h from 1345 h, just before the predicted change of the tide from flood to ebb. The pump was calibrated prior to the dye release using a stopwatch and graduated cylinder. The weather on the day of the dye release was dry with a light breeze from the north.

Dye fluorescence concentrations were measured at fixed locations using two C-FLUOR fluorometers (Turner Designs™) attached to navigation marker piles Nos. 7 and 28 and by tracking the surface dye plume with a fluorometer deployed over the side of a vessel, according to methods described by Campos et al. (2017a, 2017b). The fixed fluorometers recorded dye concentrations over 7 days and were recovered on 17 February 2021. On 9 February 2021, a preliminary survey was conducted to collect background fluorescence data and instal the submersible fluorometers.

Boat tracking of the dye plume was conducted using a C3 fluorometer to identify the shape and edges of the plume as it travelled through Sawyers Bay and main harbour channel. Dye fluorescence concentrations were tracked in surface waters during daylight hours on the afternoon of 10 February 2021 (between 1433 and 1838 h) and on the morning of 11 February 2021 (between 0933 and 1153 h). Traverses were conducted in the bay on both occasions from north to south and from east to west. The dye fluorescence readings were downloaded from each fluorometer and plotted in Excel alongside tidal depth data downloaded from the NIWA Tide Forecaster website (NIWA, 2019) for the period of the study. A five-point moving average was applied to the dye concentrations to reduce the statistical effect of outliers for graphical display, after subtracting an average background concentration. Dilution was calculated by dividing the initial concentration in the dye mixture by the concentrations recorded in seawater. Water column profiling of temperature and salinity was conducted at the dye release site, at the shellfish sampling sites in Sawyers Bay and growing area 1805 and at the two fluorometer locations using a SBE 19 plus V2 SeaCAT CTD (Sea-Bird Scientific™) (site locations are shown in Supplementary Information).

The movement (speed and direction) of surface waters within Sawyers Bay was determined by releasing four self-logging GPS drogues at four sites along the anticipated path of the dye plume just after the dye release start time (10 February 2021). Drogue positions were logged via the GPS tracklog function at 30-s intervals. Each drogue was tracked for approximately 4 h or until the drogue grounded. Following retrieval, the tracklog from each drogue GPS was downloaded to a PC and each track was converted to a GIS-compatible polyline. The data from each GPS track were then overlaid on Google Earth™ for display.

Two unmanned aerial vehicles (UAVs) (DJI Mavic Mini) equipped with GoPro cameras were used to survey the dye plume as it travelled within Sawyers Bay. Plume surveillance by UAV followed a pre-defined flight. Two unmanned aerial vehicles (UAVs) (DJI Mavic Mini) equipped with GoPro cameras were used to survey the dye plume as it travelled within Sawyers Bay. Plume surveillance by UAV followed a pre-defined flight plan and took approximately 4 h. One UAV was flown from the survey vessel, while the second UAV was flown from advantage points on the shoreline. To allow continuous surveillance of the plume, each UAV was initially flown to an altitude of approximately 200 m and placed in a hovering position above the location of the dye release. During hovering, the UAVs were moved horizontally in a north–south direction to allow visual appreciation of the plume. Each flight took <10 min, with short breaks between flights. Photographs and videos were downloaded to a PC for subsequent analysis.

The hydrographic connectivity between the Sawyers Bay PS outfall and the wider harbour areas was assessed using a particle tracking computational tool. Briefly described, the tool is a 2D fast Lagrangian particle tracking model developed using the ‘OceanTracker’ code in unstructured grids based on currents (depth-averaged) derived from a hydrodynamic model (Vennell et al., 2021). The model output produced for this study shows the trajectories of 30 particles released at the pump station outfall site simulated over a 30-day period.

### Statistical analyses

The distribution of the microbiological results and their suitability for parametric testing were assessed by probability plots and Ryan-Joiner tests. The distributions of microbiological concentrations were found to be non-normal and were log_10_-transformed prior to statistical analyses. *E. coli* concentrations below the limit of quantification (<20 MPN/100 g) were replaced by 19 MPN/100 g. After transformation, the data did not conform to normal distribution and were subsequently analysed using non-parametric tests. Descriptive statistics (minimum, maximum, geometric mean and 95% confidence interval for the mean) were calculated to summarise the microbiological data.

Kruskal–Wallis tests were calculated to compare differences in geometric mean concentrations for samples collected during baseline monitoring and emergency closures. Mann–Whitney tests were used to compare differences in geometric mean concentrations. The data were also graphically displayed by scatterplots. Tests conducted for individual sampling sites showed no differences in mean concentrations of microbiological contaminants. To increase statistical confidence in the results, data from sites A, B and C were combined and averaged for 1804, 1805 and SB sites. All statistical analyses were performed in Minitab 20™.

## Results

### Microbiological and virus results

#### Concentrations and distribution of E. coli and viral markers

In total, 218 shellfish samples were collected during the study (baseline [n = 136] and emergency closure samples [n = 82]). Overall, *E. coli* concentrations in baseline samples ranged from <20 to 1,100 MPN/100 g, while those in emergency closure samples ranged from <20 to 9,200 MPN/100 g (Table 2). The geometric mean *E. coli* concentration in emergency closure samples from growing area 1804 was significantly higher than that in baseline samples (Mann–Whitney [M-W] test; Z = −2.6; P = 0.009). Mean PMMoV concentrations (GC /gram digestive tissue) in emergency closure samples from 1805 and SB were higher than those in baseline samples (M-W tests; Z = −2.0, P = 0.043; Z = −2.2, P = 0.026, respectively) (Table 2). Mean crAssphage concentrations in emergency closure samples from 1804 and 1805 were higher than those in baseline samples (M-W tests; Z = −3.1, P = 0.002; Z = −2.9, P = 0.003, respectively). The F-RNA GII results are not included in this analysis because of the low number of samples with concentrations greater than the LoQ (200 GC/g digestive tissue) (0 baseline samples; 10 emergency closure samples).

**Table 2:** Summary statistics of microbiological concentrations in shellfish. (*) Indicates significantly higher mean in emergency pollution relative to baseline

Site ID	n	Minimum	Maximum	Lower CI	Geometric mean	Upper CI
*E. coli*						
Baseline monitoring						
1804	42	<20	490	25	32	42
1805	48	<20	490	26	32	40
SB	46	<20	1,100	54	77	109
Emergency pollution conditions						
1804	27	<20	790	39	59*	88
1805	26	<20	1,100	31	50	80
SB	29	<20	9,200	70	138	274
PMMoV						
Baseline monitoring						
1804	36	200	7,871	948	1,410	2,098
1805	41	200	15,388	1,293	2,089	3,375
SB	40	122	12,422	983	1,485	2,243
Emergency pollution conditions						
1804	25	200	43,732	1,597	3,453	7,465
1805	26	200	82,946	2,477	5,684*	13,044
SB	29	200	94,766	2,108	4,339*	8,934
CrAssphage						
Baseline monitoring						
1804	33	200	780	202	223	246
1805	45	200	1,152	247	285	328
SB	44	200	1,357	310	366	431
Emergency pollution conditions						
1804	19	200	2,133	317	454*	649
1805	24	200	5,105	452	718*	1,140
SB	27	200	6,463	427	670	1,052

n—number of samples with quantitative result

CI—confidence interval

Overall, 11.8% of baseline shellfish samples exceeded the *E. coli* limit for ‘Approved’ areas (230 MPN/100 g). This compares with 18.3% of samples that exceeded this limit during emergency growing area monitoring. Concerning the overall prevalence of viral markers (expressed as a percentage of positive samples), PMMoV and crAssphage were more prevalent than F-RNA GII. In baseline samples, the prevalence of the markers was 83% for crAssphage (113 samples), 85% for PMMoV (117 samples) and 15% for F-RNA GII (21 positive samples, all with concentrations at LoQ). In emergency closure samples, the prevalence was 85% for crAssphage (15 samples), 98% for PMMoV (80 samples) and 26% for F-RNA GII (36 positive samples, 10 samples > LoQ).

The microbiological concentrations were also assessed by growing area and by type of sample in reference to the limit for ‘Approved’ growing area and to the concentrations exceeding the LoQ of the PCR method. The results show a higher percentage of samples with *E. coli* concentrations exceeding the ‘Approved’ limit during emergency closure monitoring relative to baseline monitoring (Table 3). The percentage of samples with viral concentrations greater than the LoQ was also higher in emergency closure samples for crAssphage (sites 1804 and 1805) and for PMMoV (sites 1805 and SB) (Table 3). No samples had F-RNA concentrations greater than the LoQ.

**Table 3:** Number and percentage of samples exceeding the *E. coli* limit for ‘Approved’ growing area and samples with viral concentrations above the limit of quantification of the PCR method

	*E. coli*	F-RNA GII	CrAssphage	PMMoV
Site ID	Number and percentage of samples >230 MPN/100 g	Number and percentage of samples >200 copies/g		
Baseline monitoring				
1804	2 (4.8)	0 (0)	6 (18.2)	29 (80.6)
1805	2 (4.2)	0 (0)	22 (51.2)	31 (75.6)
SB	12 (26.1)	0 (0)	31 (70.5)	31 (77.5)
Emergency growing area closures				
1804	3 (11.1)	0 (0)	12 (63.2)	20 (80.0)
1805	3 (11.5)	0 (0)	16 (66.7)	22 (84.6)
SB	9 (31.0)	0 (0)	16 (59.3)	24 (82.8)

#### Variation of microbiological concentrations as a function of time since last spill and distance from the wastewater outfall

The variation of microbiological contaminants following the wastewater overflows was investigated for individual spill events. Scatterplots of *E. coli* and viral marker results relative to time since the spill events are shown in Supplementary Information S1. *E. coli* concentrations at sites in growing area 1804 reduced significantly (*p* = 0.018) over time following spill event 1. CrAssphage concentrations in growing area 1805 also reduced significantly (*p* = 0.038) over time following spill event 1. However, the levels of explained variance of these statistically significant models were low (R^2^ = 52% and R^2^ = 41%, respectively), which is typical of microbiological data in environmental studies. Following spill event 3, crAssphage concentrations in growing areas 1804, 1805 and SB increased over time (*p* = 0.001, R^2^ = 66%; *p* = 0.000, R^2^ = 81%; *p* = 0.007, 49%; respectively), which is counterintuitive. It is likely that catchment diffuse sources of crAssphage (i.e. stormwater discharges, surface water run-off) determined these results, but we were not able to confirm this assumption. None of the other models were statistically significant. Of note is that, of the 17 samples taken in growing areas 1804 and 1805 within 2–3 days from spill end times, only three samples had *E. coli* concentrations greater than 230 MPN/100 g (‘Approved’ limit), which indicates that bacterial concentrations in the shellfish flesh reduced rapidly following the wastewater overflows.

The variation of microbiological contaminants as a function of the distance from the pump station outfall per spill event is also shown in Supplementary Information S2. None of the linear regression models were statistically significant, except that the model representing the variation of *E. coli* at sites in growing area 1804 following spill event 2 (*p* = 0.04; R^2^ = 61%).

### Dye study and particle tracking modelling results

#### Dye fluorescence measurements taken by fixed fluorometers and boat tracking

**Fig. 2:**
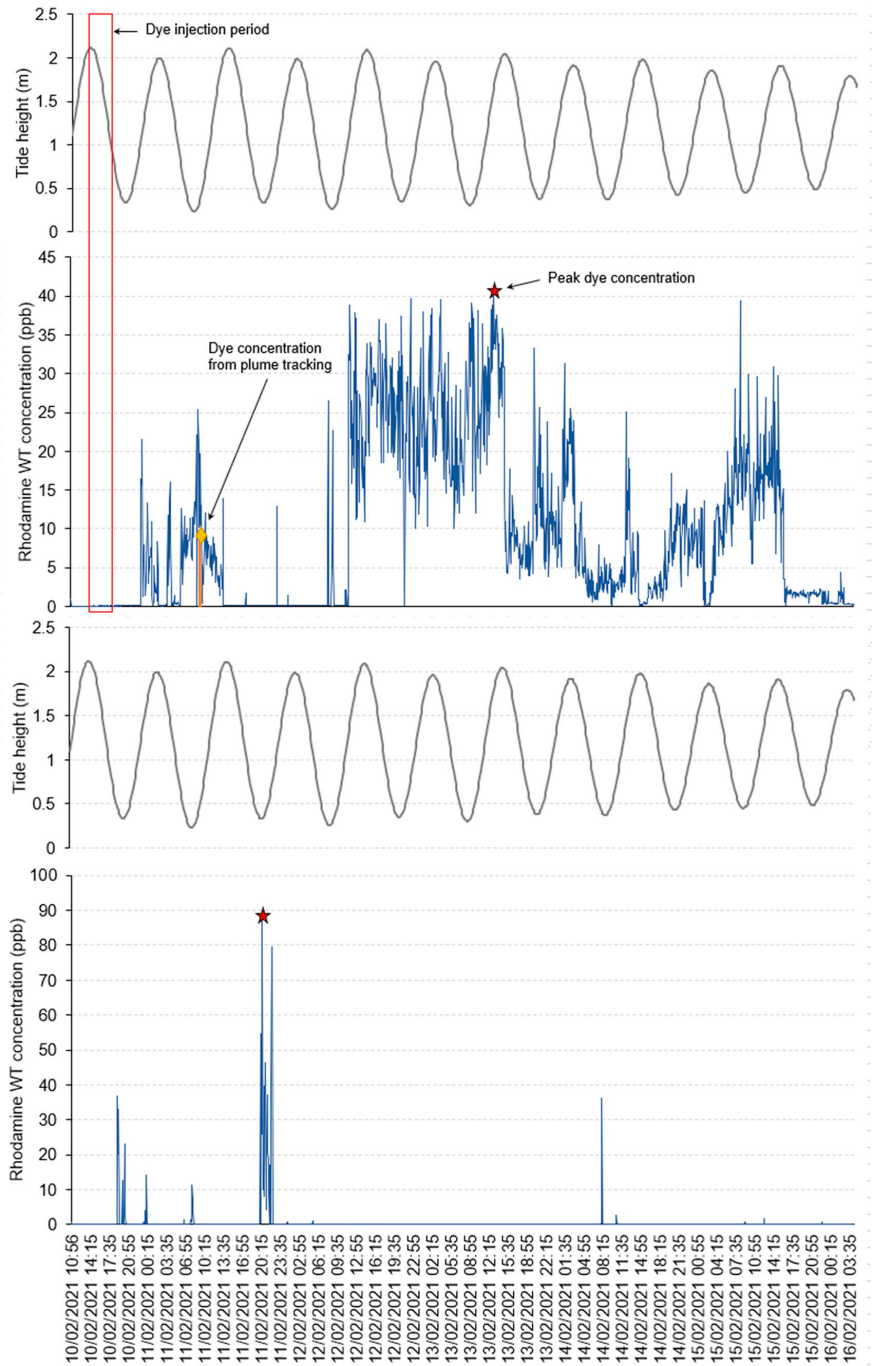
Dye fluorescence results obtained by the fixed fluorometers at Pile 7 (A) and Pile 28 (B)

A total of 1,690 dye fluorescence readings were collected by the fixed fluorometers. Continuous fluorescence measurements at the fixed monitoring sites (Piles 7 and 28; site locations shown in Fig. 1) together with water levels in Sawyers Bay are shown in Fig. 2. At Pile 7 (mouth of Sawyers Bay), the first patch of dye (approximately 16 ppb) was recorded on 10 February 2021 at 2345 h (low water +3 h). A second larger patch of dye (26.5 ppb) was firstly recorded on 12 February at 0925 h (low water) and remained in the bay over subsequent tidal cycles with peak levels of 36–40 ppb until 13 February at 1700 h (high water). The peak dye concentration was 40 ppb on 13 February at 1505 h (high water −1.5 h). Further smaller patches of dye were recorded until 15 February at 1940 h. From this time, fluorescence levels just above background levels were recorded until the fluorometers were recovered on 16 February 2021. The observed long residence time of the dye plume indicates that wastewater discharged from the Sawyers Bay PS is slowly flushed away from the bay.

At Pile 28, the fluorometer recorded only a few very small patches of dye with peak levels of 37 ppb on 10 February 2021 at 1900 h, 88 ppb on 11 February at 2025 h and 78 ppb at 2205 h, and 36 ppb on 14 February at 2005 h. All these peak levels were recorded at or just before low water times. During all other times over the deployment period, the fluorometer recorded fluorescence concentrations at or just above background levels. The dye profile at this station suggests that the area was impacted by the dye plume to a very small extent. However, the possibility that the fluorometer was not well positioned to represent the impact of the dye plume on growing area 1804 cannot be discounted.

**Fig. 3:**
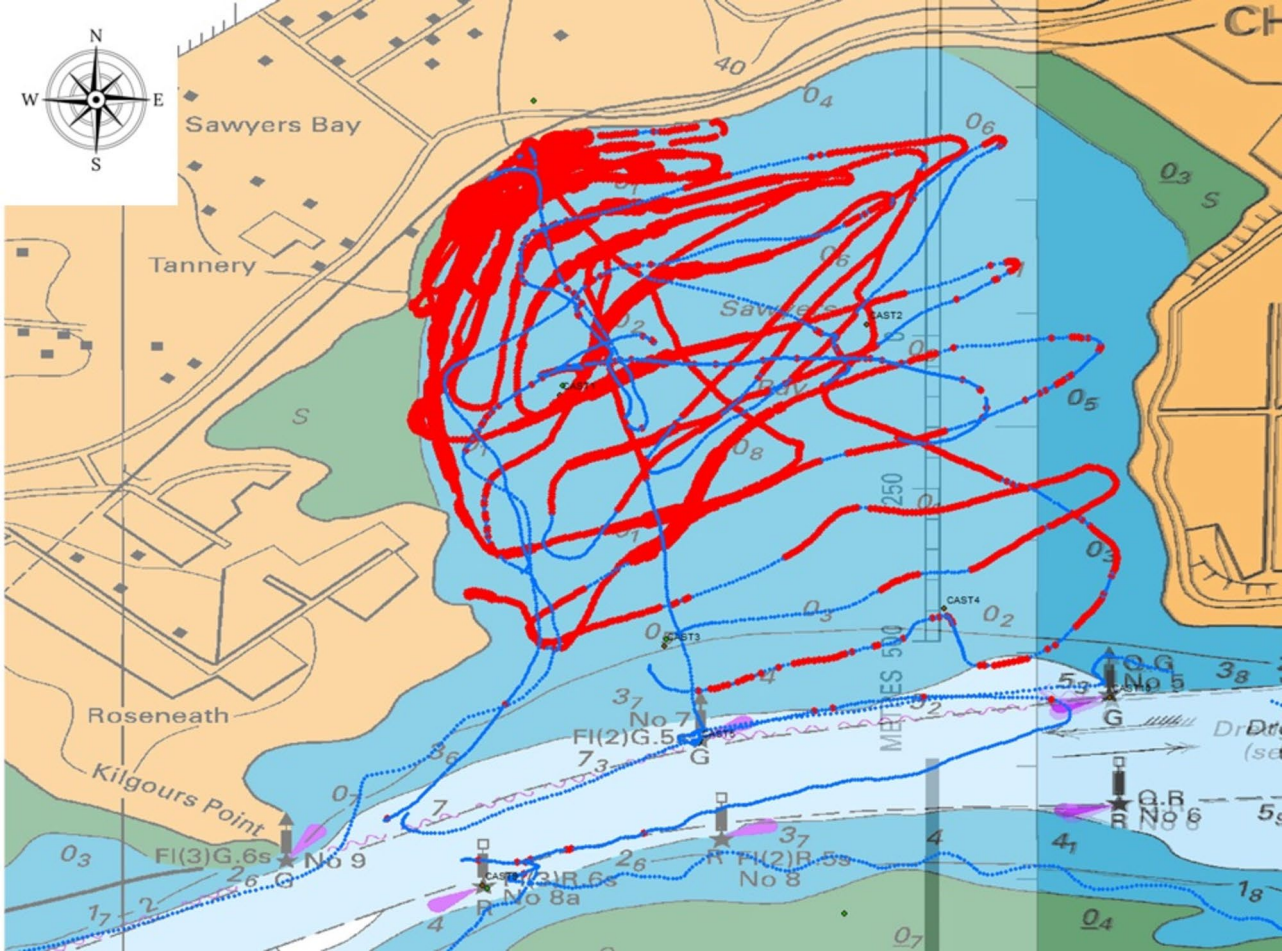
Dye fluorescence results obtained by the tracking fluorometer in Sawyers Bay

A total of 6,772 dye fluorescence readings were collected by the boat-tracking fluorometer. The average fluorescence during the boat-tracking period was 24 ppb. The fluorescence measurements accumulated over the two tracking periods (10 February 2021 [14:33–18:38] and 11 February 2021 [09:33–11:53]) in Sawyers Bay are shown in Fig. 3. The results show reducing gradients of dye from the dye release site to the mouth of the bay and from west to east, which indicates that, in the event of a spill from the Sawyers Bay PS outfall, the wastewater would impact mainly the western part of the bay. The blue line crossing the bay in a north–south direction shows the traverse conducted just before the dye release when there was no dye in the bay. The blue lines along the southern edge of the navigation channel (Piles 8a, 8 and 6) show that no dye impacted growing area 1805 during the tracking period.

A small quantity of dye (peak level of 12.6 ppb) was recorded near Pile 7 on 11 February at 1020 h, which is consistent with that recorded by the fixed fluorometer (10.7 ppb) (Figs. 2 and 3). Trace concentrations of dye were recorded at the mouth of Sawyers Bay, indicating some incursion of dye into the channel. However, dye was not detected around the edges of growing area 1805 during the tracking period (three traverses conducted 1.5 tidal cycles post-dye release), suggesting that the dye plume advected around Port Chalmers Peninsula (shown on the right in Fig. 3) on the ebb tide. The dye tracking results also confirm the long residence time of the waters in Sawyers Bay suggested by the fixed fluorometer results.

A time series of aerial photographs taken from 1405 h (20 min after the dye release) to 1745 h (4 h after release) is shown in Supplementary Information (S3). An area of concentrated dye is visible extending from the release site alongside the pump station outfall into the centre of the bay. Patches of dye were observed to contact the western shores. These dye patches later merged to form a large plume that spread across the western part of the bay.

#### Drogue tracks and time of travel

**Fig. 4:**
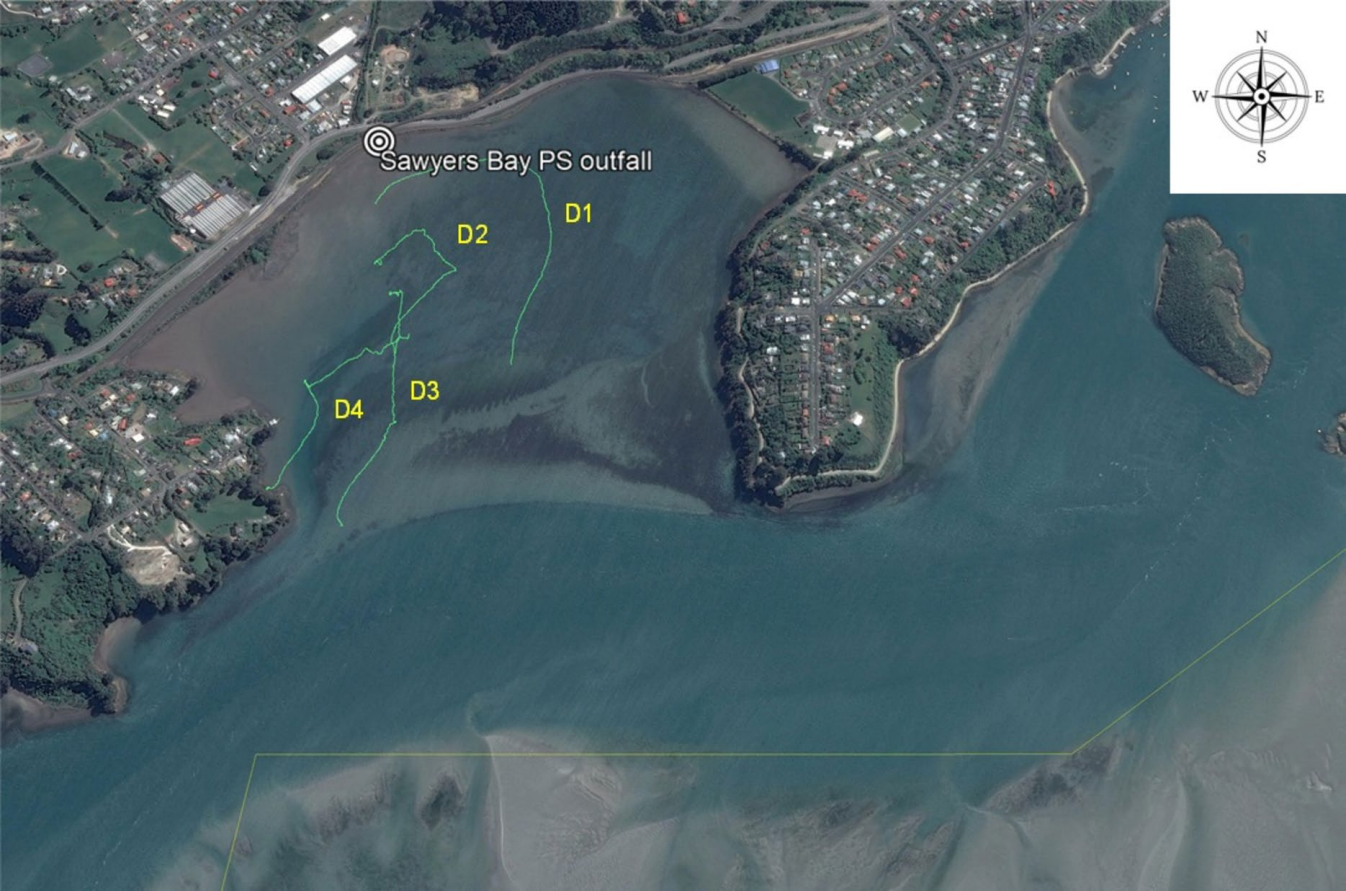
Drogue tracks (shown as green lines) in Sawyers Bay. N.B. All drogues were released at inshore sites and travelled towards the mouth of the bay

Four drogues (labelled as D1–D4 in Fig. 4) were released at sites along the anticipated path of the dye plume within Sawyers Bay. The drogues released at the most inshore sites (D1, D2) travelled in a clockwise direction, while the remaining drogues (D3, D4) travelled in a northeast-southwest direction (Fig. 4). All drogues were recovered at the mouth of Sawyers Bay. The drogue tracks are generally consistent with the boat-tracking dye fluorescence results illustrated in Fig. 3.

The travel time of a wastewater discharge from the Sawyers Bay PS outfall to the shellfish growing areas was estimated from the drogue tracks and the dye fluorescence readings at Pile 7 on the ebb tide of 10 February 2021. Based on the drogue release and recovery times and distances travelled, the mean velocities estimated for the drogues ranged from 0.20 to 0.28 km/h (Table 4). The mean time of travel as indicated by the drogue study was 0.25 km/h. Dye released at 1110 h was first detected by the submersible fluorometer at Pile 7 at 2345 h. When comparing the initial and final drogue tracking times with the travel times suggested by the dye study, it is evident that the dye plume travelled at a much slower rate (0.08 km/h) than the drogues. The drogues used are very light, and the differences in travel time can be attributed to strong water density effects in Sawyers Bay slowing down the movement of dye entrapped near the surface because of the large volume of fresh water entering the bay. Wind strength was very low at the time of the drogue study (<6 knots; light breeze), and therefore, it is unlikely that wind-driven currents influenced these results.

**Table 4:** Estimated times of travel of wastewater contamination in Sawyers Bay based on drogue tracks and dye fluorescence concentrations

Method	Time (initial)	Time (final)	Distance travelled (m)	Travel time (km/h)
Drogue ID				
D1	13:50	17:31	1,014	0.28
D2	13:53	17:50	789	0.20
D3	13:54	17:33	901	0.25
D4	13:56	17:21	885	0.26
				Mean = 0.25
Dye plume			1,000	0.08

The movement of wastewater contamination in the study area is determined by tides and wind-driven currents. On the afternoon of 10 February 2021, northerly winds with an average velocity of 11 km/h pushed the dye from the release site to the mouth of Sawyers Bay. Winds of ≤11 km/h occur only 5% of the time in the study area (Bell et al., 2009). Northerly winds are typical, but westerly winds also occur with high frequency. These westerly winds are likely to prolong the residence time of the wastewater within the bay. Under stronger northerly winds (typical in the study area), faster movement of wastewater contamination within Sawyers Bay can be expected. Based on the drogue travel times, it was estimated that there is a period of 6 h (notification time) for wastewater contamination from the Sawyers Bay PS discharge to reach the edge of the shellfish growing area 1805. A longer travel time was estimated from the dye results (16 h for growing area 1804). However, this estimate is unrealistic because wastewater travels faster in the navigation channel than in Sawyers Bay, and these travel time differences were not accounted for in the calculations. Consequently, wastewater would reach growing area 1804 sooner than 16 h.

#### Water temperature and salinity

**Fig. 5:**
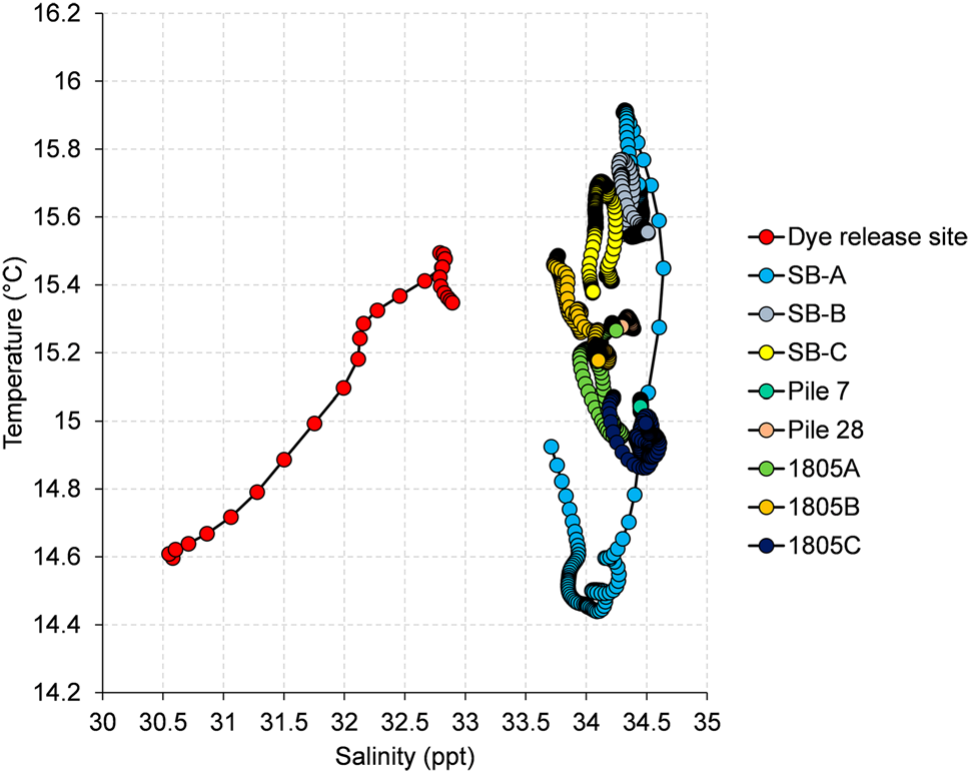
Temperature-salinity diagram for nine sites in the study area

A temperature-salinity (T-S) diagram is shown in Fig. 5. Sites ‘dye release’ and SB-A had a distinct T-S profile compared to that at sites in the shipping channel (piles 7 and 28) and shellfish growing area. The largest salinity range was detected at the dye release site (30.5–32.9 ppt), while the largest temperature range was detected at site SB-A (14.4–15.9 °C). The smallest T-S variations were found at Pile 7 (34.4–34.5 ppt; 15.0–15.1 °C) and Pile 28 (34.3–34.4 ppt; 15.2–15.3 °C).

#### Particle dispersion

The particle tracks are shown as blue lines in Supplementary Information S4. The red crosses show the connectivity points on the shoreline. All particles released from the Sawyers Bay PS outfall travelled towards the upper harbour. The results show a very small connectivity between Sawyers Bay and the shellfish growing area 1805 and no connectivity with the growing area 1804. Of the 30 particles released from the Sawyers Bay PS outfall, only three connected with the north shore. Twenty-four particles (80%) connected with the stretch of shoreline between Raynbirds Bay and Turnbulls Bay.

### Relationships between microbiological results and wastewater dilution

**Fig. 6:**
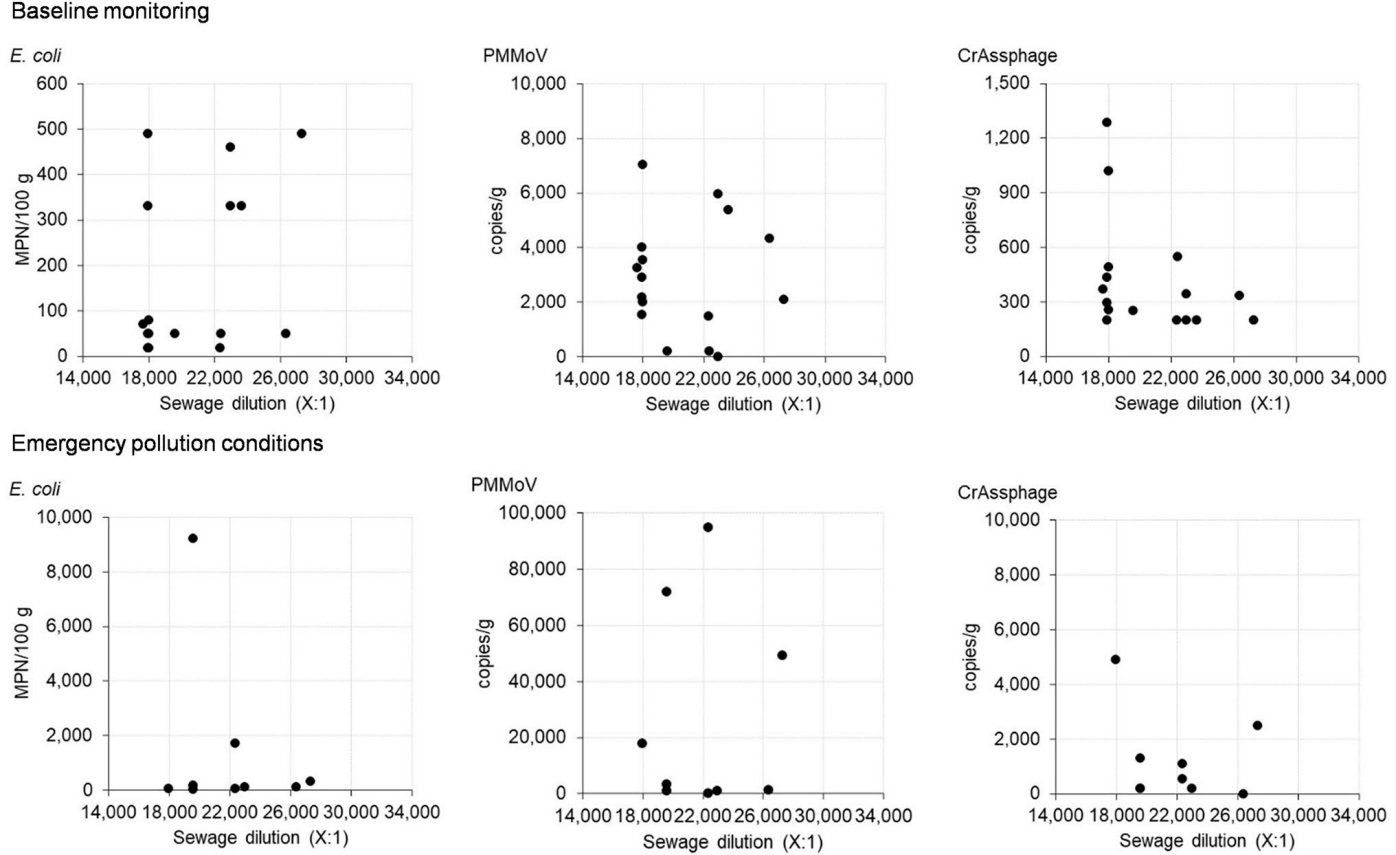
Scatterplots of concentrations of E. coli and viral indicators in shellfish and wastewater dilution at site SB-C. NB. Results shown are from SB-C site only (nearest to Pile 7 fluorometer)

The maximum instantaneous dilution of wastewater at site SB-C (near Pile 7) was calculated using the dye tracing results to investigate the relationships between this parameter and the microbiological concentrations in shellfish. Figure 6 shows scatterplots for *E. coli* and two viral markers (crAssphage, PMMoV) for baseline and emergency closure samples. The baseline sampling plots represent a scenario of normal harvesting activity following a small volume wastewater discharge that has not exceeded the growing area management criterion (150 m^3^). Data for F-RNA GII are not presented because few samples were positive for this marker (three baseline samples and one emergency closure sample).

In baseline samples, there was an apparent reduction of crAssphage/PMMoV concentrations with increasing wastewater dilution (Fig. 6). However, linear regression analysis showed that the relationships are not statistically significant. In emergency closure samples, the results also showed no relationship between the variables, which indicates that wastewater dilution is not a suitable parameter for predicting microbiological contamination in the study area.

## Discussion

This study determined the prevalence and distribution of *E. coli*, norovirus GI and GII, and three viral markers (F-RNA GII, crAssphage, PMMoV) in shellfish growing areas located in an urban tidal inlet during periods of normal harvesting and following wastewater overflows. It also investigated the dispersion, dilution and time of travel of wastewater contamination and the relationships between the microbiological contaminants and time since last spill, distance between the overflow site and the growing areas and wastewater dilution, to inform decisions on closure/reopening of harvesting activity following spills.

Unlike more highly populated countries, NZ has a large animal-to-human ratio and most shellfish growing areas are remote from large population centres (Greening & McCoubrey, 2010). Consequently, the risk of contamination of shellfish growing areas with domestic wastewater is perceived to be lower in NZ than that in western European countries. However, a nationally representative survey of norovirus in shellfish production areas has not been carried out. A study that included testing from non-commercial sites impacted by pollution sources showed high norovirus prevalence (50% positive samples) with concentrations >1,000 GC/g digestive tissue detected in 25% of positive samples (Hewitt et al., 2019). Previous studies found 30% of samples positive for norovirus in the Bay of Islands (Greening, 2007), 32% of samples positive for norovirus GII in Tauranga Harbour (Scholes et al., 2009) and 38–71% of samples also positive for norovirus GII at coastal sites in Dunedin (Greening & Lewis, 2007) (all sites were near polluted urban areas). In the present study, norovirus was not detected in any of the 218 samples tested. This is likely associated with the small catchment population contributing wastewater in the study area (approximately 4,000) and/or the lack of norovirus in the community around the sampling dates. While gastroenteritis outbreaks are notifiable, there is likely underreporting of cases and outbreaks. During the study period (January 2021, February 2022, and July 2022), only one norovirus outbreak in the city of Dunedin was reported with samples submitted to ESR for norovirus surveillance purposes (in February 2022) (pers. comm. Norovirus Reference Laboratory).

Several studies in NZ and overseas have assessed the spatial and temporal distribution of norovirus in coastal environments (Brake et al., 2017; Campos et al., 2015; Green et al., 2022; Greening & Lewis, 2007; Winterbourn et al., 2016). Overall, these studies show that viral levels decline with distance and time from sewage sources. One study undertaken in a shallow estuary impacted by a high number of wastewater spills (162 events with duration varying from a few minutes to 38 days) reported small reductions (0.6 log_10_) in norovirus concentrations in oysters spanning approximately 10 km from the discharge point over 128 days (Campos et al., 2015). Another study in an open coast deep water environment impacted by a discharge of secondary-treated effluent reported a 0.2 log_10_ reduction in norovirus GII concentrations in caged mussels at a site 2 km from the outfall over the 30 days of cage deployment (Winterbourn et al., 2016). In contrast to the areas previously studied, Otago Harbour is not impacted by a continuous wastewater discharge and overflows to tidal waters occur infrequently. Water movement in this harbour is strongly influenced by the deep navigation channel. Data from acoustic Doppler current profiler deployments show that depth-averaged water current velocities in the main shipping channel can be 2 × higher or more than those in the flanking sandbanks (Bell et al., 2009). The particle tracking model output illustrates this with a high number of particles following the path of the channel in the upper harbour. On the day of the dye release, Pile 7 and Pile 28 had smaller T-S ranges than those at sites in Sawyers Bay and growing area 1805 indicating waters with different physico-chemical characteristics in the channel. Taken together, these results indicate that the shipping channel reduces exposure of the shellfish growing areas to contamination from Sawyers Bay and potentially other sources in the upper harbour. This conclusion is supported by the very small hydrographic connectivity between the dye release site and the shellfish growing areas observed in the dye study, the lower mean *E. coli* concentrations at the 1804 and 1805 sites relative to those at Sawyers Bay sites, and the lack of relationships between microbiological contamination and wastewater dilution. Furthermore, the sand banks are exposed during low tide, while the shellfish cages were immersed at all times, which may also explain the difference in mean *E. coli* levels between the growing area and Sawyers Bay sites.

Previous studies demonstrated that wastewater dilution combined with viral indicator monitoring can be used to manage norovirus contamination following wastewater spills (Campos et al., 2017a, 2017b; Goblick et al., 2011). For the three wastewater spills monitored in the present study, no significant evidence was found of microbiological impact on the growing areas. This suggests that, for smaller volume spills from the Sawyers Bay PS (≤327 m^3^), some reduction in the closure period for impacts from this source on growing areas 1804 and 1805 could be considered in the future. Five lines of evidence were found to support this claim:

- No norovirus was detected in any of the shellfish samples collected following the wastewater spills, suggesting that the virus was not present in the local community during the study period.
- 82% of the shellfish samples collected within 2–3 days post-spills had *E. coli* concentrations below the ‘Approved’ limit (230 MPN/100 g), which indicates rapid bacterial decay in the growing areas.
- The concentrations of viral markers in emergency closure samples were not dissimilar from those in baseline when plotted against time since spill and distance from the wastewater discharge.
- Samples collected post-spills with *E. coli* concentrations greater than 230 MPN/100 g had no F-RNA GII concentrations above the LoQ (200 GC/g).
- The mean concentrations of viral markers (PMMoV and crAssphage) in emergency pollution condition samples were lower than average levels found in other commercial shellfish growing areas in NZ (during closed harvest periods) (Gyawali et al., 2021).

The potential for microbiological contamination from Sawyers Bay to impact growing area 1804 cannot be discounted given the small patch of dye detected by the fixed fluorometer at Pile 28. To determine appropriate closure periods following spills in Otago Harbour and other shellfish growing areas with similar characteristics, confirmatory viral testing of shellfish samples should be combined with an assessment of contaminant transport in the area affected (through field and/or modelling studies). Ideally, viral testing would include the pathogen(s) of concern. Site-specific norovirus contamination data are preferred over data reported in the overseas literature given the differences in viral seasonality reported for different climatic regions (Ahmed et al., 2013). A contaminant transport study for wastewater spills could consider the release of dye (as a ‘slug’ or continuously and proportionally to the flow of the discharge) and/or drogues and visual tracking of the dye cloud/drogues in the impacted area (Kilpatrick & Cobb, 1985; FAO & WHO, 2021). Ideally, such study would be undertaken at the time of the sanitary survey and the results reviewed periodically to develop a model of contamination fate. Freshwater flows and wind-driven water circulation have a significant effect on the dispersion and dilution of wastewater contamination. Consequently, it may be appropriate to undertake multiple dye releases during different weather conditions. To determine appropriate closure periods following spills, it is important to determine the dispersive characteristics of wastewater and estimate time of travel for all pollution sources of concern (FAO & WHO, 2021). A fit-for-purpose risk communication procedure and monitoring preparedness assessment similar to that recommended by Kirby et al. (2014) for oil spills would allow flexibility and efficiency in the decision-making process and the deployment of appropriate resources.

Detection and quantification of all viruses of concern following a wastewater overflow is not practical in the context of regulatory shellfish growing area monitoring. A risk assessment jointly undertaken by the US FDA and Health Canada supports the use of F-RNA bacteriophage for shellfish growing area management (Pouillot et al., 2022). F-RNA bacteriophages behave similarly to norovirus in the marine environment (Hodgson et al., 2017) and are recommended by the Interstate Shellfish Sanitation Conference for reopening growing areas following wastewater overflows (USFDA & ISSC, 2019). Other potentially useful indicators proposed in the literature are crAssphage (Farkas et al., 2019) and PMMoV (Gyawali et al., 2019; Symonds et al., 2017). Source specificity, sensitivity and prevalence have been evaluated for these indicators in NZ using animal faecal material, untreated wastewater, treated wastewater and shellfish (Gyawali et al., 2021). The Gyawali et al. study indicated that while crAssphage and PMMoV have greater sensitivity for predicting norovirus, F-RNA GII has greater predictive specificity. The study concluded that testing of crAssphage and F-RNA GII, by PCR, provides a more robust estimation of norovirus presence than either viral marker alone. The present study did not support or refute these results in the context of wastewater spill effects because norovirus was not detected in any samples. However, despite the elevated concentrations of viral markers detected at some sites post-spills, the concentrations were similar to background concentrations detected in other shellfish growing areas in the absence of wastewater spills (Gyawali et al., 2021).

## Conclusions

- The combination of dye tracing, drogue tracking, UAV surveillance, particle tracking modelling and viral testing of shellfish samples resulted in successful characterisation of the microbiological impacts of small volume wastewater overflows from Sawyers Bay PS on the shellfish growing areas in Otago Harbour.
- Norovirus was not detected in any of the samples collected during baseline and emergency growing area closures. In combination with the low concentrations of F-RNA bacteriophage GII, PMMoV and crAssphage detected in the samples (typical of background contamination), the results indicate a negligible risk of viral contamination of human origin in the Otago Harbour shellfish growing areas during the monitoring period. Sampling undertaken over 21 months provided a suitable timeframe to develop a good understanding of viral prevalence in these shellfish growing areas.
- The long residence time of the waters in Sawyers Bay combined with the strong water flows in the main harbour channel mitigate the risk of viral contamination on the growing areas from small volume spills from the Sawyers Bay PS outfall.
- The study indicated that small volume spills do not significantly impact the Otago Harbour growing areas and that a reduction to the mandatory 28-day closure period could be considered in the future.
- Flexibility in the procedures for closure/reopening growing areas impacted by spills is recommended, particularly for short duration/small volume spills and when norovirus is not present in the community, to ensure that microbiological risk is proportionate to commercial interests.

### Supplementary Information

Below is the link to the electronic supplementary material.

Supplementary file1 (DOCX 21559 KB)
