# Supplementary material for: Study of Shellfish Growing Area During Normal Harvesting Periods and Following Wastewater Overflows in an Urban Estuary With Complex Hydrography
Source: Food Environ Virol. 2024 Feb 8;16(1):79–96. doi: 10.1007/s12560-023-09579-8 (PMC10963581; doi:10.1007/s12560-023-09579-8)
Supplement: Supplementary file 1 — Supplementary file1 (DOCX 21559 KB) [file 12560_2023_9579_MOESM1_ESM.docx]

Supplementary Information

S1. Microbiological concentrations as a function of time since last spill

| Spill event 1 |
| --- |
| 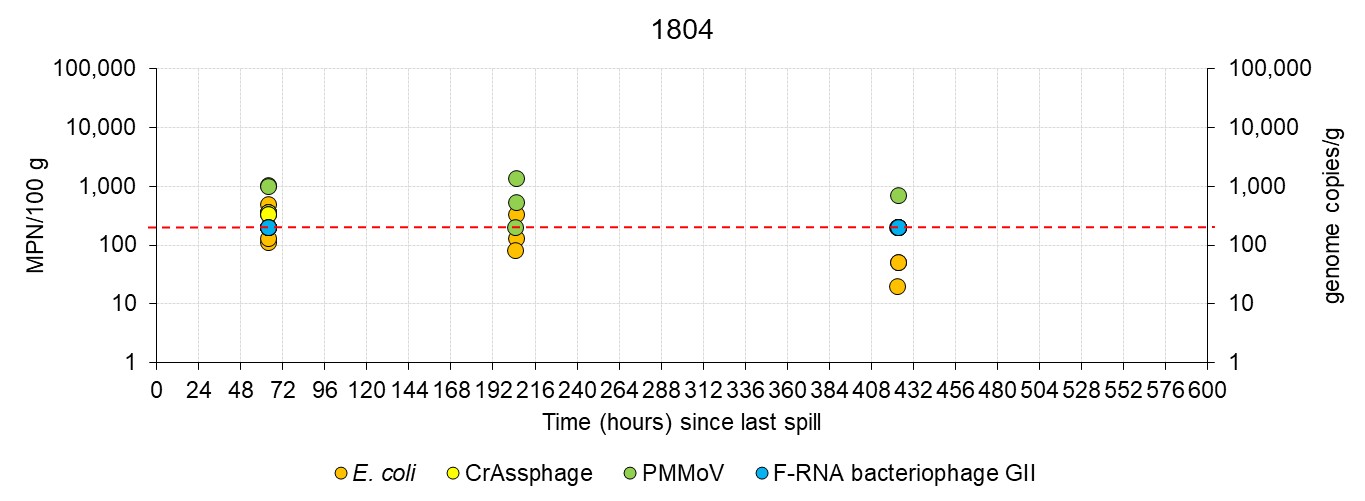 |
| 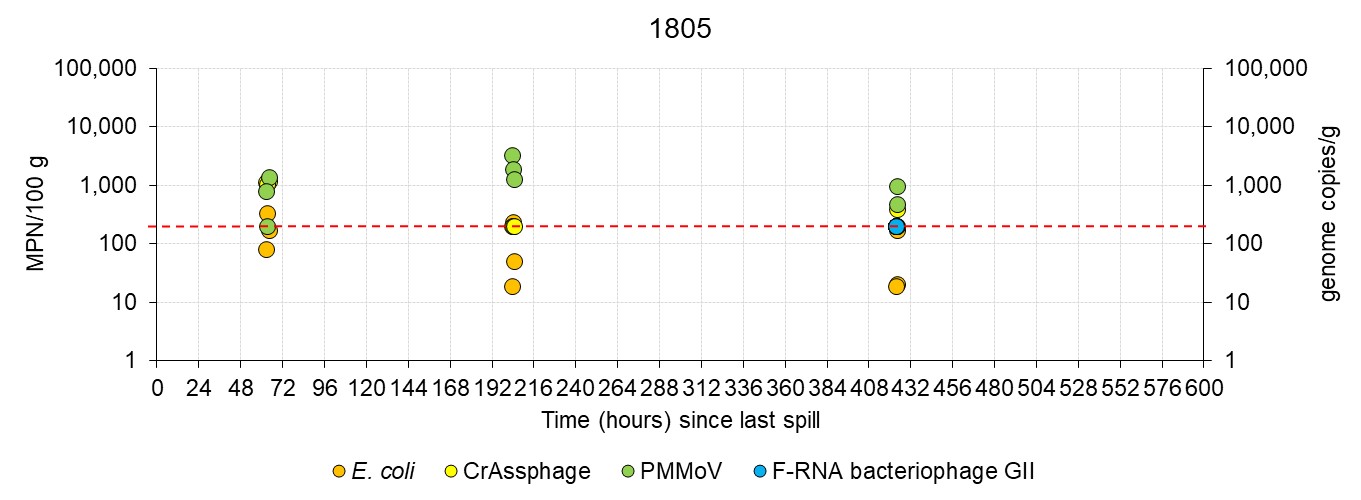 |
| 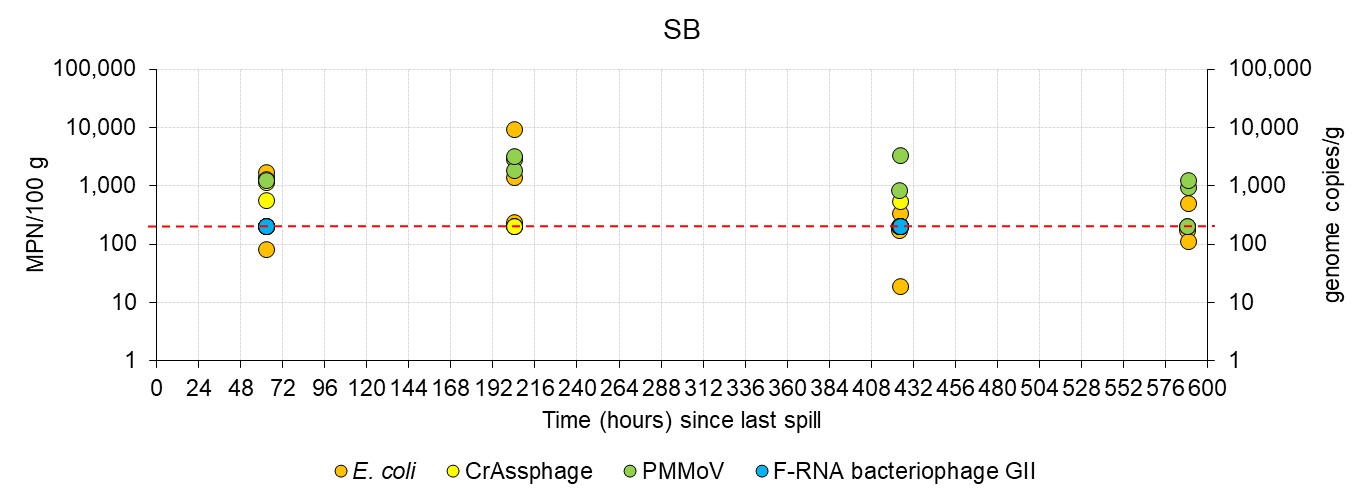 |

| Spill event 2 |
| --- |
| 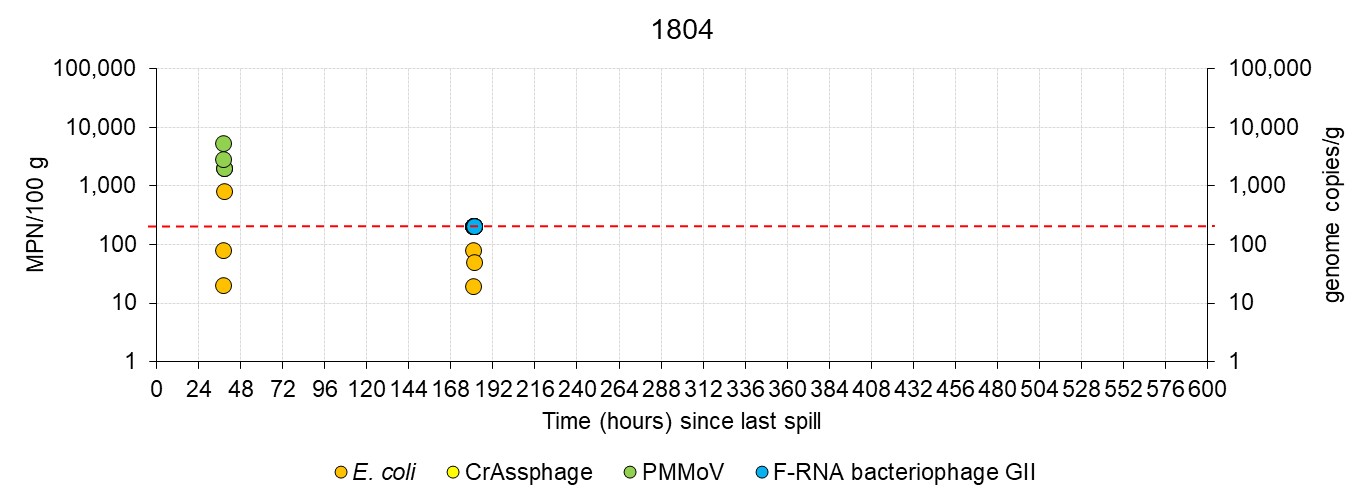 |
| 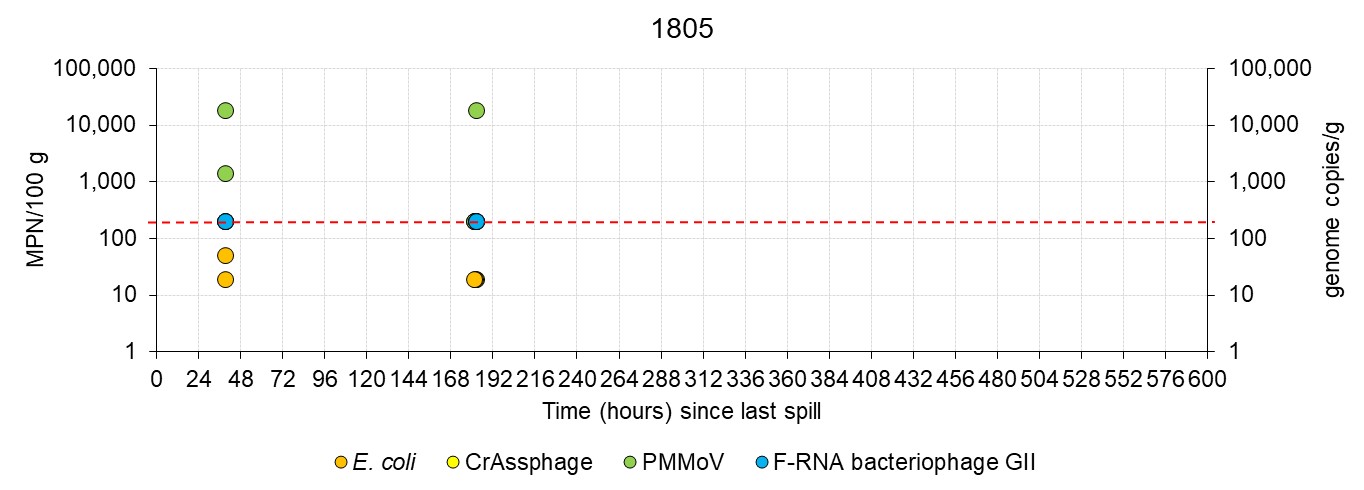 |
| 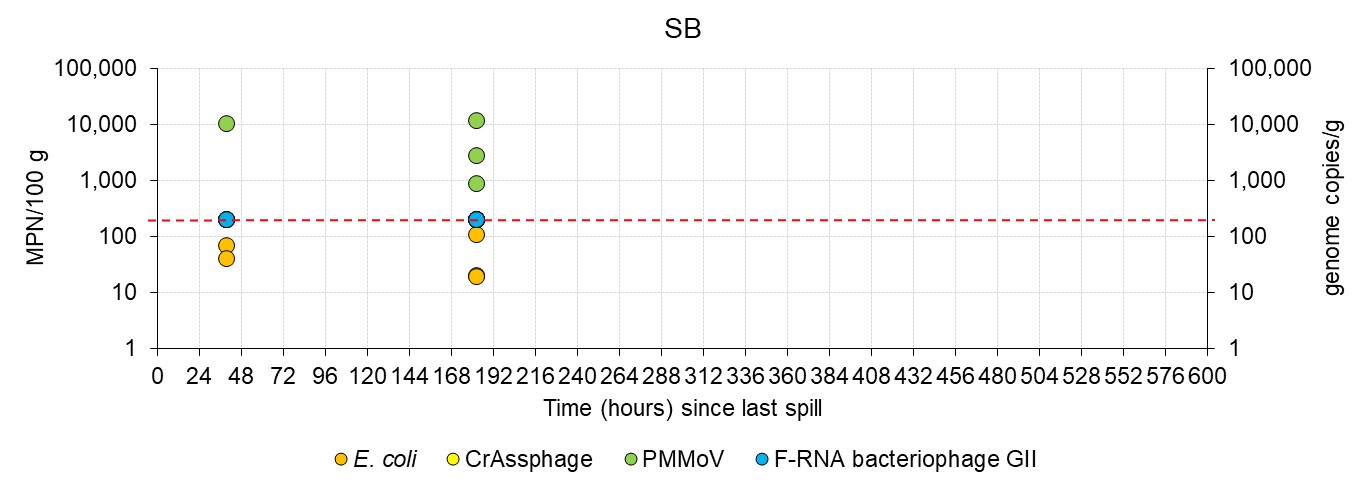 |

| Spill event 3 |
| --- |
| 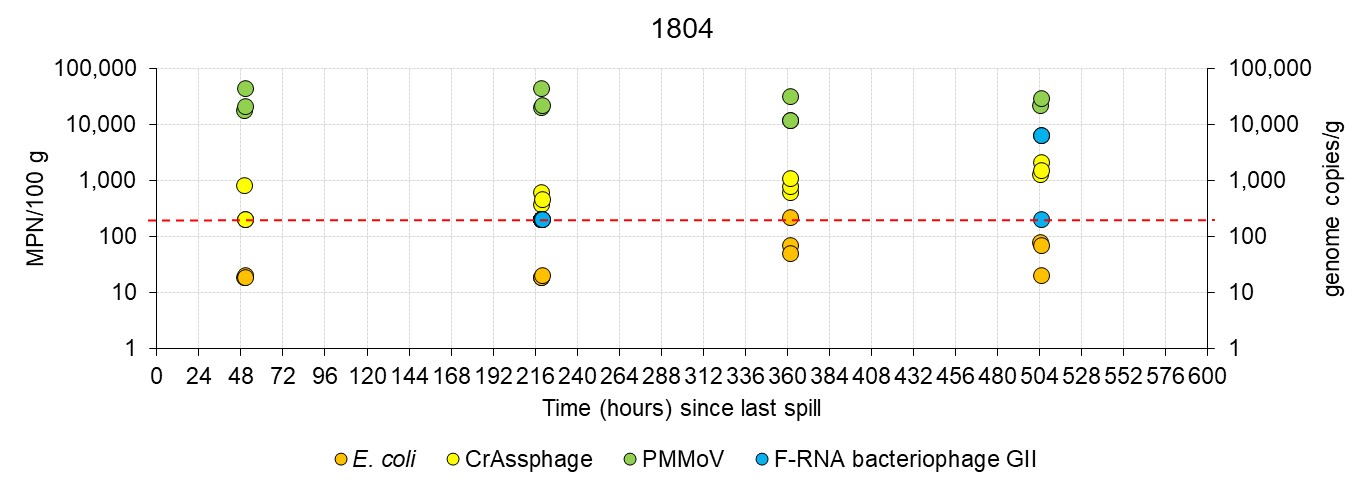 |
| 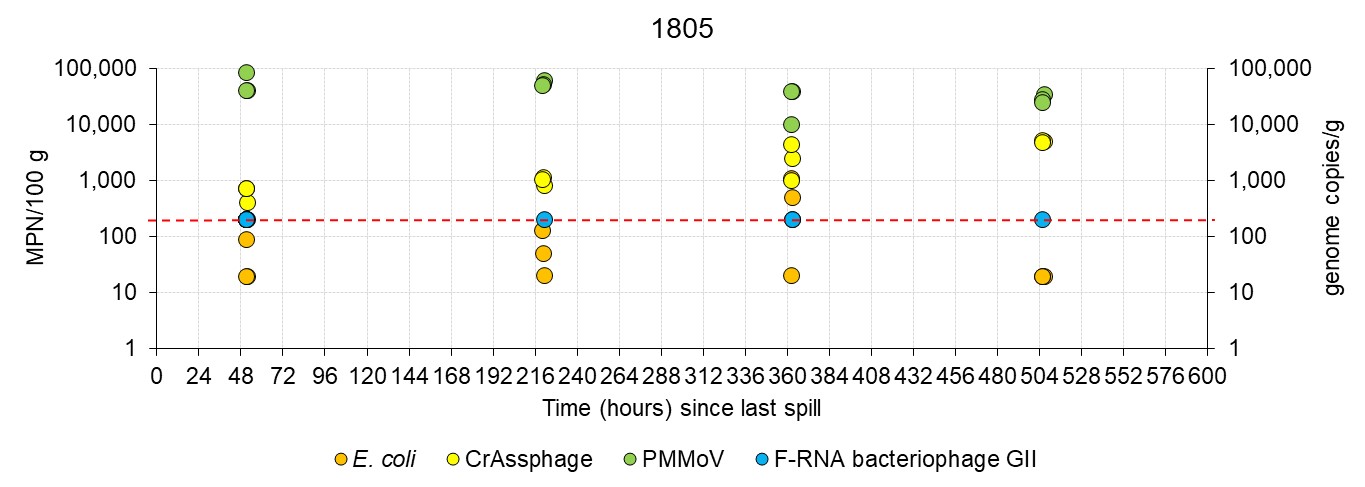 |
| 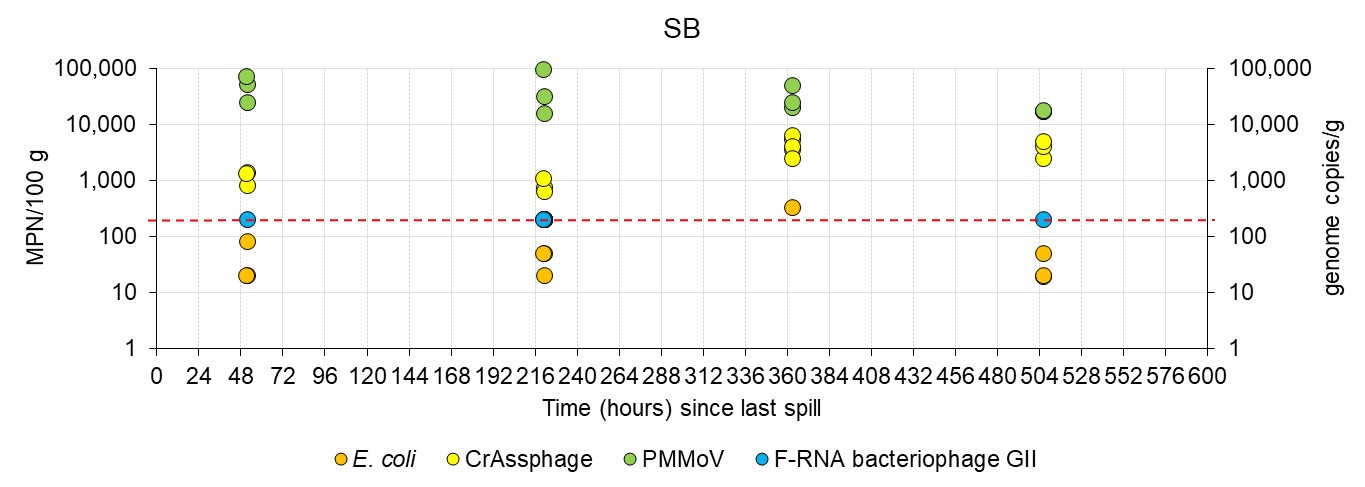 |

S2. Microbiological concentrations as a function of distance to the pump station outfall

| Spill event 1 |
| --- |
| 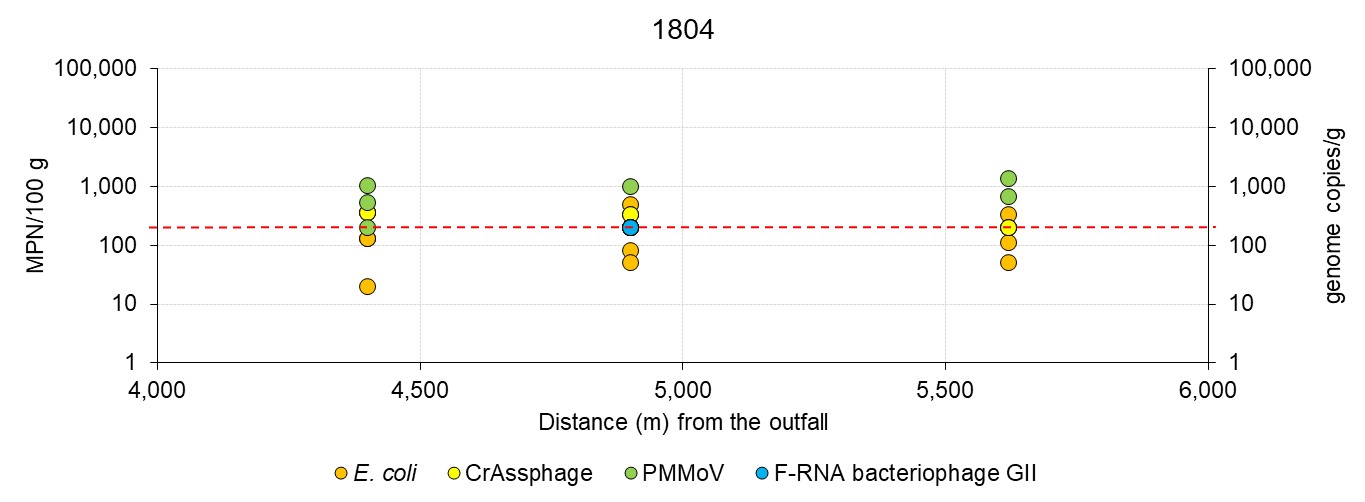 |
| 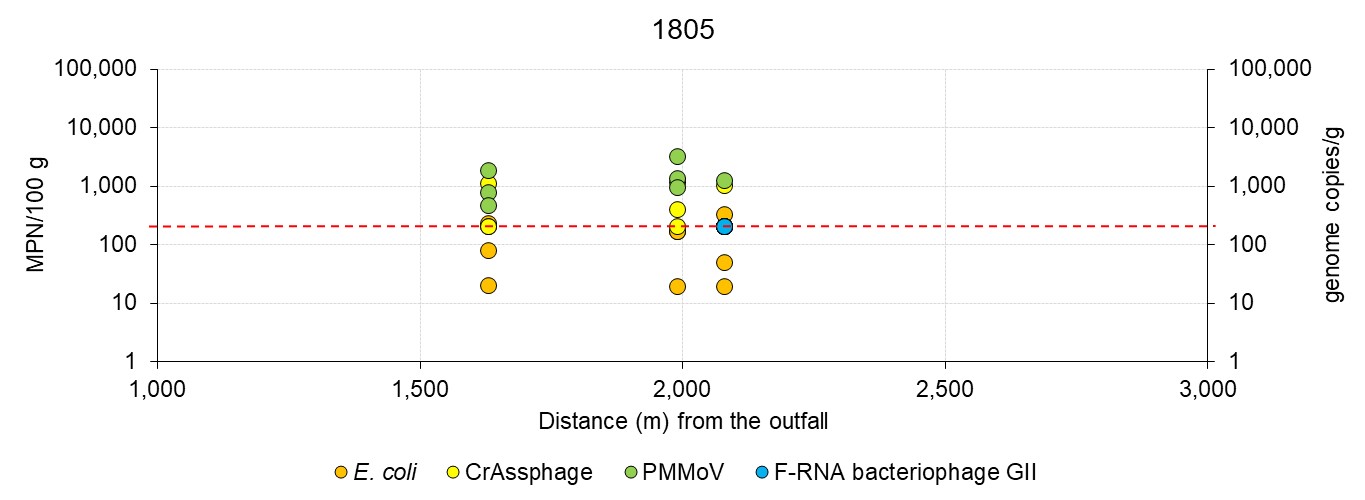 |
| 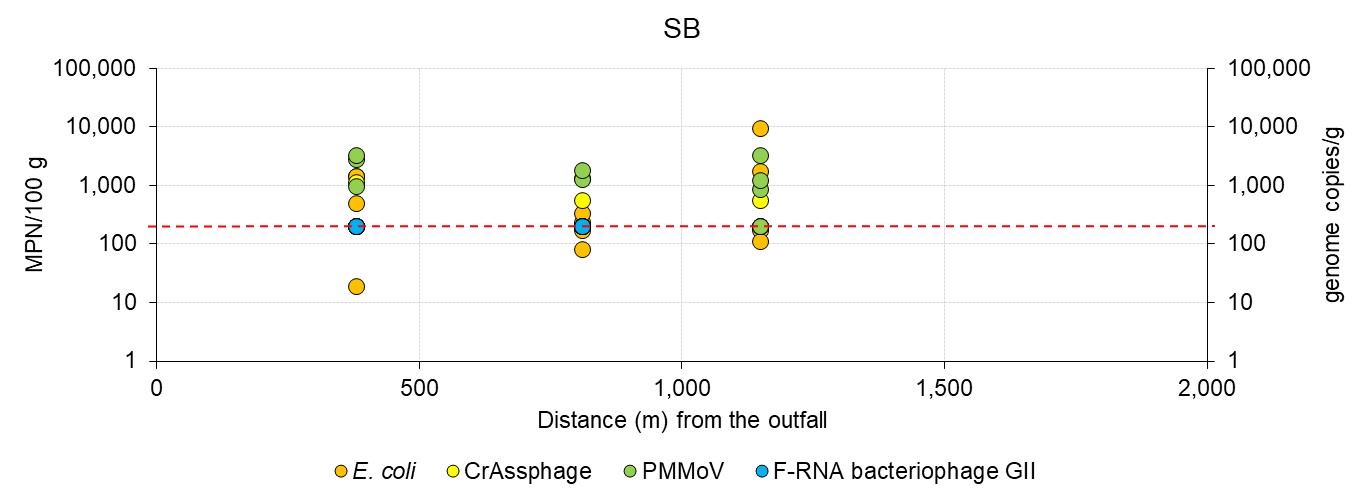 |

| Spill event 2 |
| --- |
| 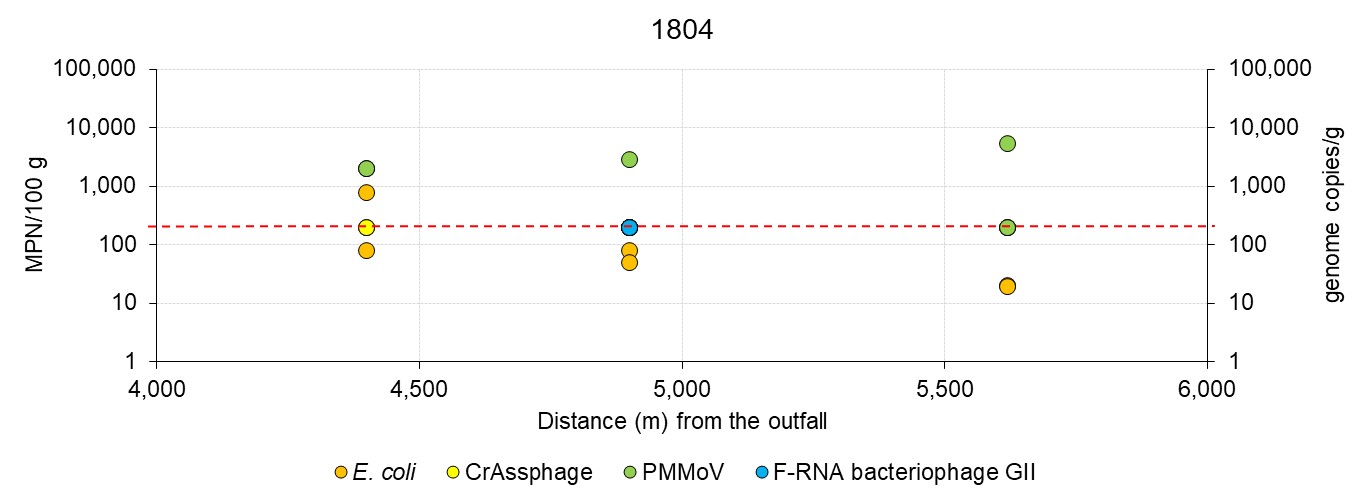 |
| 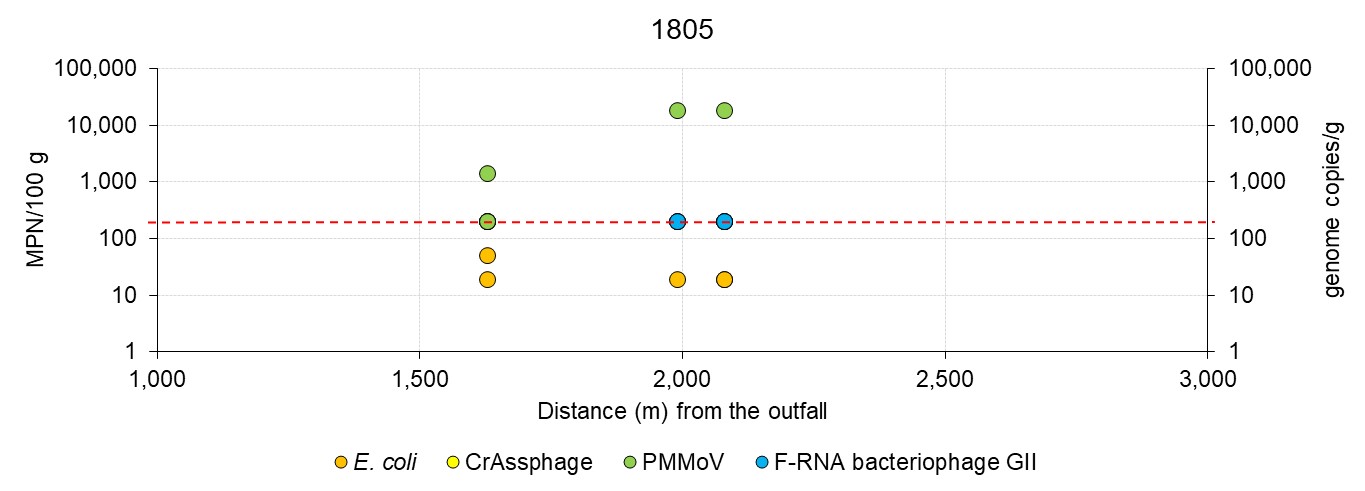 |
| 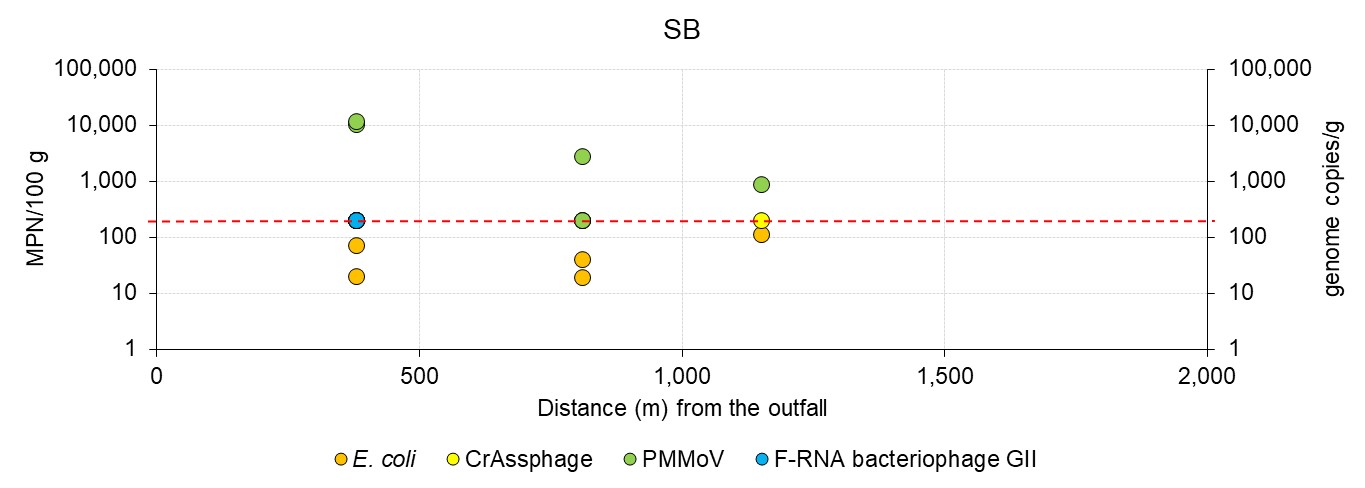 |

| Spill event 3 |
| --- |
| 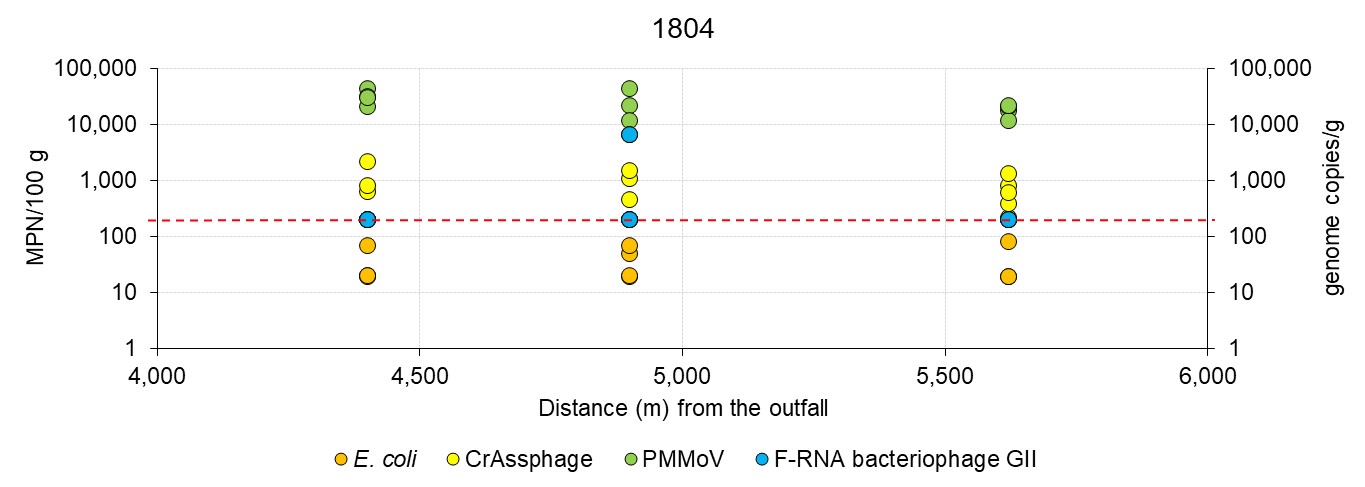 |
| 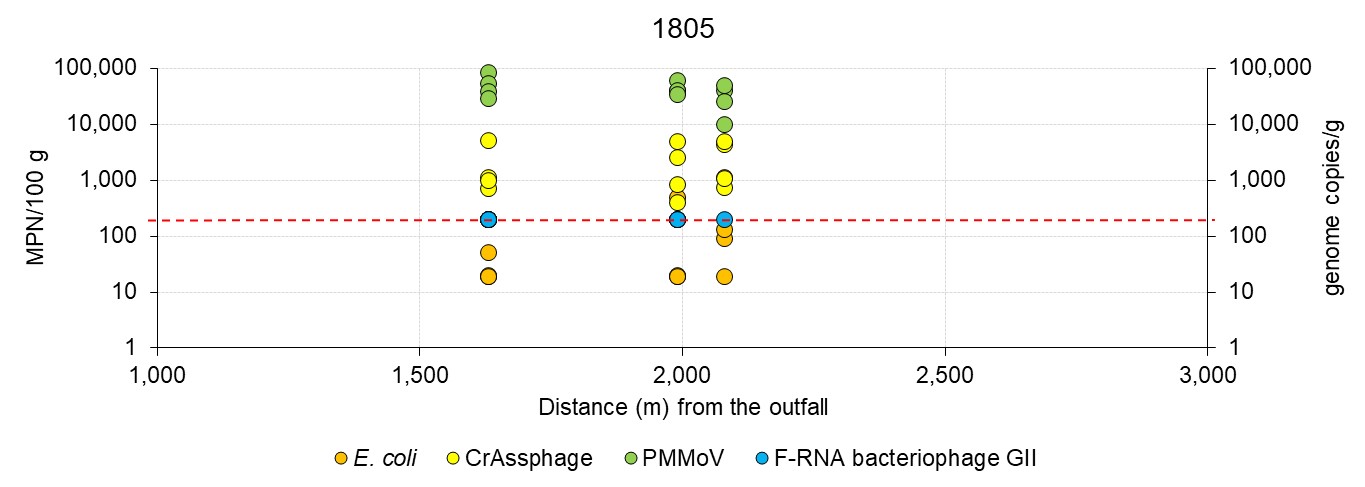 |
| 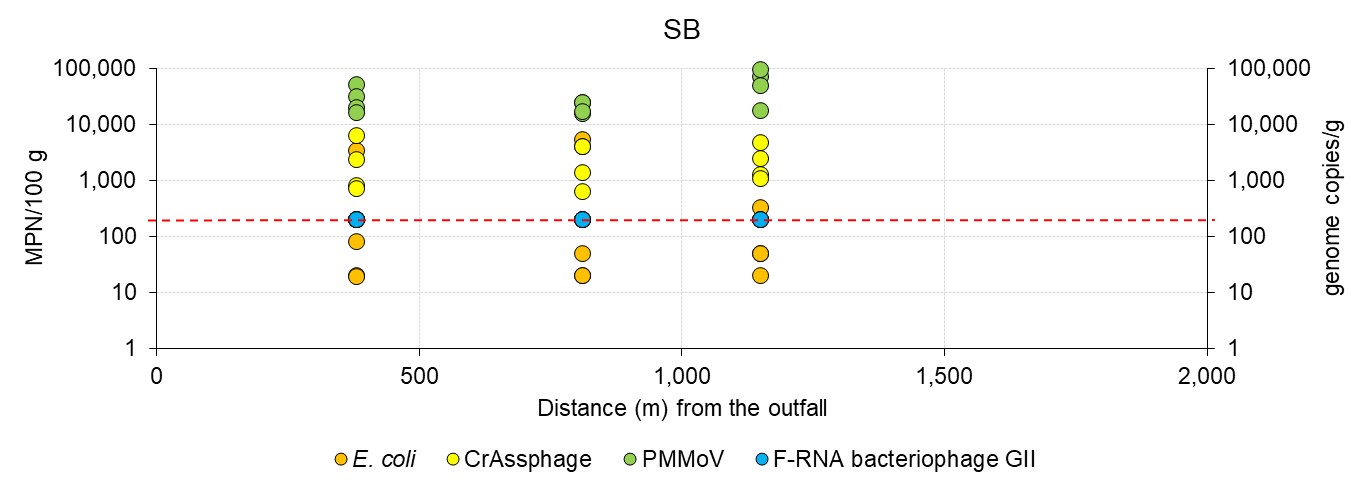 |

S3. Aerial photographs of dye patches in Sawyers Bay. Times of photographs taken: A-1405 h; B-1420 h; C-1505 h; D-1536 h; E-1621 h; F-1745 h.

| A | B |
| --- | --- |
| 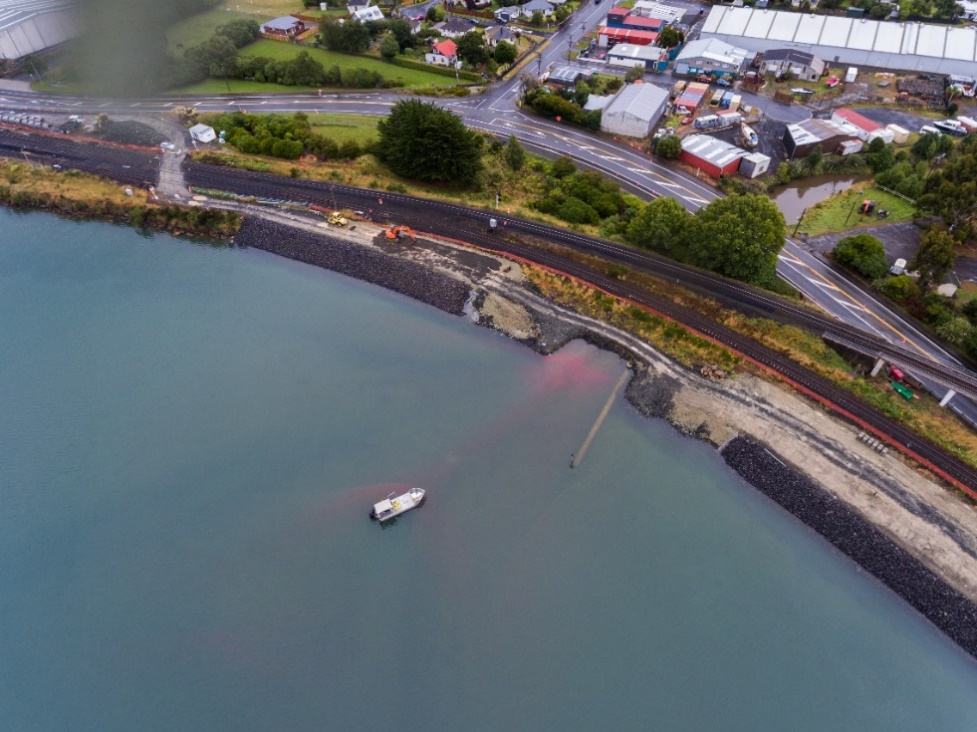 | 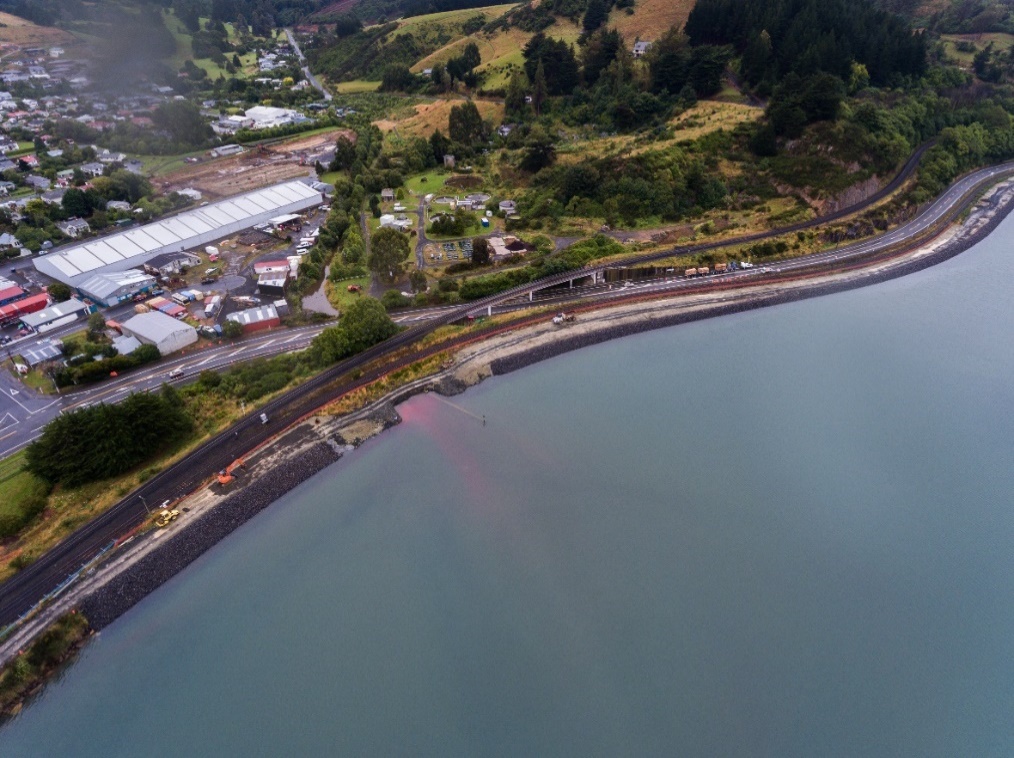 |
| C | D |
| 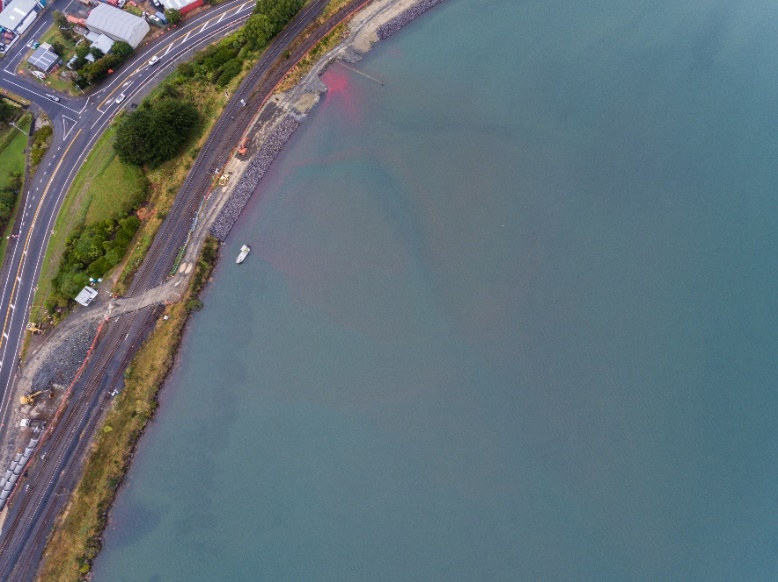 | 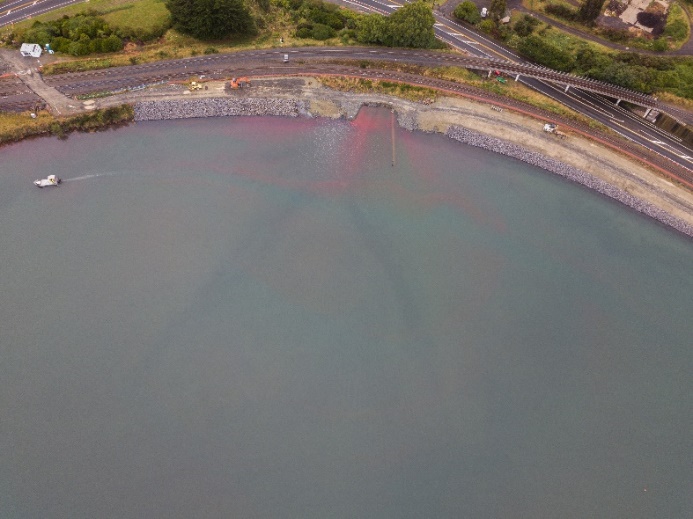 |
| E | F |
| 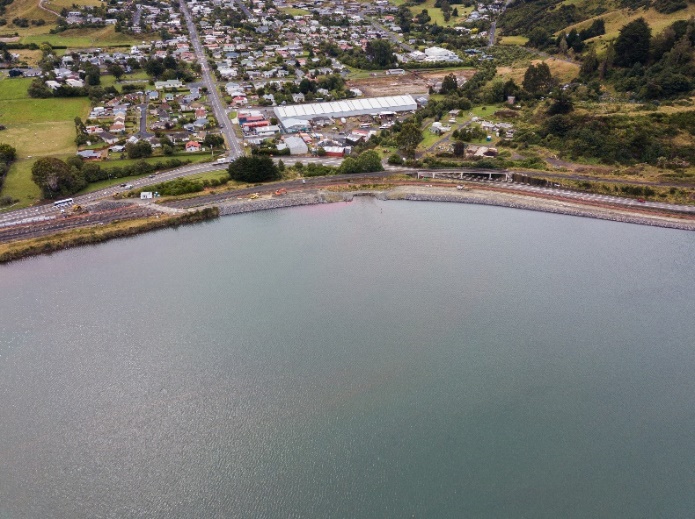 | 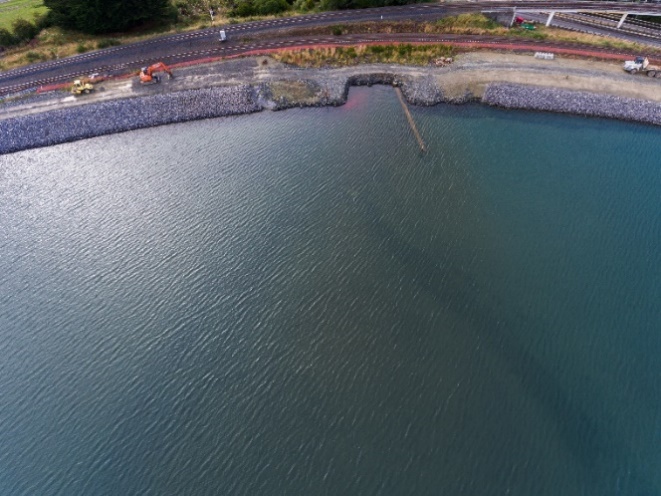 |

S4. Results of particle tracking modelling showing the trajectories of 30 particles (red crosses) released from the Sawyers Bay PS outfall over a 30-day model simulation period.
